# Archaeogenetics reconstructs demography and extreme parental consanguinity in a Bronze Age community from Southern Italy

**DOI:** 10.1038/s42003-025-09194-2

**Published:** 2025-12-15

**Authors:** Francesco Fontani, Felice Larocca, Elisabetta Cilli, Rocco Iacovera, Adam Jon Andrews, Adriana Latorre, Fabiola Arena, Rossella Veneziano, Lucio Calcagnile, Gianluca Quarta, Harald Ringbauer, Philipp W. Stockhammer, Johannes Krause, Donata Luiselli, Alissa Mittnik

**Affiliations:** 1 Max Planck Harvard Research Center for the Archaeoscience of the Ancient Mediterranean, Leipzig, Germany; 2 Department of Cultural Heritage, University of Bologna, Ravenna, Italy; 3 Department of Archaeogenetics, Max Planck Institute for Evolutionary Anthropology, Leipzig, Germany; 4 Speleo-Archaeological Research Centre “Enzo dei Medici”, Roseto Capo Spulico (CS), Italy; 5 Speleo-Archaeological Research Group, University of Bari “Aldo Moro”, Bari, Italy; 6 Clinical Genetics Unit, Department of Women’s and Children’s Health, University of Padova, Padova, Italy; 7 Section for Marine Biology, Norwegian Institute of Water Research, Oslo, Norway; 8 Department of Biological, Geological and Environmental Sciences, University of Bologna, Ravenna, Italy; 9 Department of Biomedical Sciences and Surgical Specialties, University of Ferrara, Ferrara, Italy; 10 Department of Humanities Research and Innovation, University of Bari “Aldo Moro”, Bari, Italy; 11 CEDAD - CEntro di DAtazione e Diagnostica, Department of Mathematics and Physics “Ennio de Giorgi”, University of Salento, Lecce, Italy; 12 Department of Human Evolutionary Biology, Harvard University, 11 Divinity Avenue, Cambridge, MA USA; 13 Institute for Pre- and Protohistoric Archaeology and Archaeology of the Roman Provinces, Ludwig Maximilian University, München, Germany; 14 Department of Genetics, Harvard Medical School, 77 Avenue Louis Pasteur, Boston, MA USA; 15 Max Planck—Harvard Research Center for the Archaeoscience of the Ancient Mediterranean, 11 Divinity Avenue, Cambridge, MA USA

**Keywords:** Archaeology, Consanguinity, Population genetics

## Abstract

Given its geographic location and unique history of contacts and migrations, Calabria is a core region to investigate the genetic traces of some of the numerous prehistoric demographic events in the Central Mediterranean. However, little is known regarding the ancient populations of the region before Greek colonization, reflecting gaps in the archaeological knowledge of the territory and scarcity of genetic data. Here, we analysed genome-wide data from the Middle Bronze Age site of Grotta della Monaca (1780-1380 ca. BCE) to fill these gaps and decipher funerary practices, social organization, biological kinship ties, and demographic shifts in Southern Italy during the Bronze Age. The community shows closer genetic affinity to Early Bronze Age Sicilians than to contemporaneous populations from the Italian peninsula. However, unlike contemporary Sicilian individuals, they lack eastern genetic influences, suggesting distinct ancestral trajectories and interaction networks among Bronze Age populations. Further, we suggest that burial practices were structured according to the sex and kinship relationships of the deceased. To the best of our knowledge, our data showcase the first case reported in archaeological literature of a parent-offspring incestuous union, an extreme case that we attempt to frame into the demographic landscape of prehistoric communities of Bronze Age Southern Italy.

## Abstract (web summary)

Ancient human DNA from Grotta della Monaca in reveals ancestry patterns, kinship-based burial practices, and evidence of a parent-offspring union in Bronze Age southern Italy.

## Introduction

Calabria is the southernmost region of the Italian peninsula, bordered by the Ionian Sea to the east and south, and the Tyrrhenian Sea to the west. The region has a complex history of recent and ancient migrations, which have considerably shaped the genetic structure of its inhabitants. The Hellenic colonization of the 8th century BCE brought drastic demographic and cultural changes to Southern Italy, as Greek settlers founded coastal cities in Magna Graecia. Between the 6th and 11th centuries CE, Byzantine migrations further reinforced Greek linguistic and cultural elements, traces of which survive today in minority communities^1,2^. Later, during the 15th-century, Albanian refugees fleeing Ottoman expansion settled in Calabria, forming the Arbërisht-speaking ethnolinguistic minority still present nowadays^3^. Occitan-speaking enclaves like present-day Guardia Piemontese also emerged from migrations from Southern France in the late Middle Ages^4^. All these waves of migration have left a lasting imprint on the region’s linguistic and genetic landscape^5,6^. Large-scale genomic studies on modern Calabrian inhabitants suggest genetic continuity with populations from the Eastern Mediterranean, reflecting long-term demographic interactions with populations enriched in Caucasus and Levantine ancestry^6^. In particular, modern Calabrians exhibit notable genetic affinity with present-day populations from southeastern Peloponnese^7^, a signal likely rooted in the Greek colonization of Magna Graecia. Additional contribution of Levantine and Caucasian origin can be traced back to the Neolithic period^8^, suggesting that Calabrians, as other Southern Italians, retain early genetic signatures associated with the spread of agriculture. This Neolithic gene pool – originating in the Southern Caucasus and Anatolia – likely entered Southern Italy via Mediterranean maritime routes^5^. Supporting this interpretation, archaeological findings indicate an earlier Neolithic human presence in the region compared to Central and Northern Italy^9^. By 6000 BCE, Northern Calabria witnessed one of the earliest waves of peopling, evident at the Neolithic site of Favella di Corigliano, one of Italy’s oldest agricultural villages^10^. The region’s wealth in minerals likely spurred colonization in both northern and southern areas of the region since prehistoric times. Extensive mining activities in various caves across Calabria provide some of the oldest evidence of mineral exploitation in European prehistory^11–13^. Moreover, its strategic position as a gateway to Sicily, separated by a narrow 3.1 km-wide strait, facilitated the establishment of trade networks, especially in obsidian. From the northern side, Calabria experienced a long-lasting cultural influence from Apulia, where many prehistoric archaeological horizons, such as the Neolithic Diana culture and the Chalcolithic Laterza facies, emerged and spread^14,15^.

Despite the relatively well-documented historical migrations in the region, the demography of the prehistoric indigenous people of Calabria remains largely unknown.

**Fig. 1:**
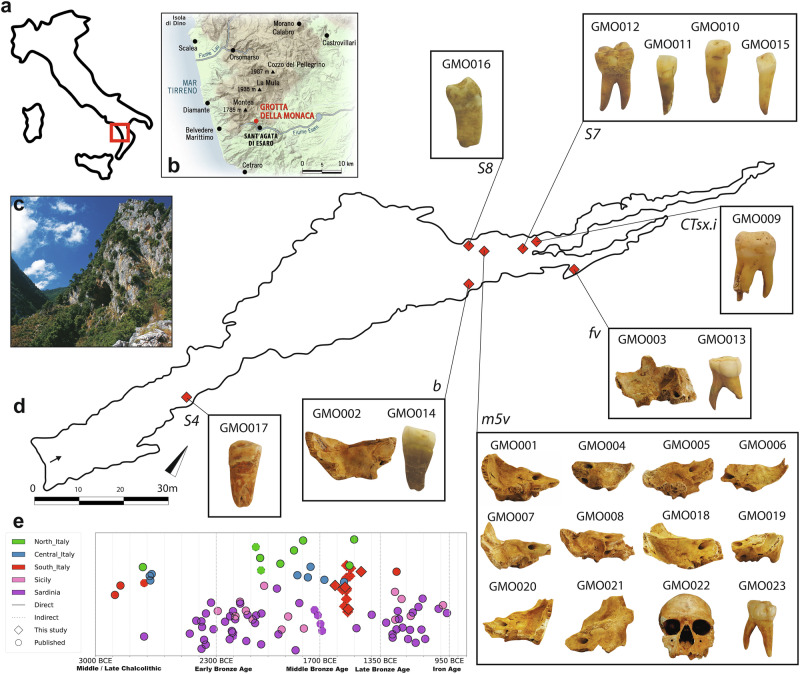
Location, archaeological context and chronological framework of Grotta della Monaca. **a** The red square broadly indicates the geographic location of Northern Calabria within the Italian peninsula; **b** the zoom in shows the location of Grotta della Monaca in Northern Calabria. Graphic elaboration by Francesco Breglia; **c** entrance of the cave, viewed from below the limestone massif. Photograph by Felice Larocca; **d** planimetry of Grotta della Monaca. The entrance in the southern sector is marked with a black arrow. Red diamonds indicate the areas of the site where scattered human remains were found. The samples analysed in this study are enclosed in black boxes, with the name of each sector indicated in italic; **e** timeline of Middle Chalcolithic to Late Bronze Age palaeogenetic data from the Italian peninsula (*n* = 144, data taken from the Allen Ancient DNA Resource repository v62, freely available at https://dataverse.harvard.edu/dataverse/reich_lab), with new samples from Grotta della Monaca shown as red diamonds. Only individuals with reliable chronological attribution, based on radiocarbon dating (Direct) or archaeogenetic evidence (Indirect), are displayed. A random jitter was applied to avoid overplotting on the y-axis.

The scant presence of settlements, especially along the Tyrrhenian coast compared to the more densely populated Ionian side, could be attributed to the unfavourable geography of the Northeastern side, primarily mountainous, which limited the development of large populations and productive lands. The inhomogeneity and hardness of the territory, in fact, posed challenges to the establishment of long-lasting, large-sized settlements in inland northwestern Calabria^16^. Furthermore, prehistoric research in Calabria has been largely dependent on survey-based investigations, resulting in conspicuous gaps in stratigraphic data, chronological frameworks, and contextual analysis, which limit our ability to reconstruct settlement patterns and cultural developments^14^. Consequently, the prehistoric demography and social dynamics of the region leading up to the end of the Bronze Age remain understudied. Calabria’s strategic position in the Mediterranean and its unique history of interaction and migration make the region an ideal case study for deciphering the genetic imprints of prehistoric demographic events in Southern Italy. However, a critical gap persists in palaeogenetic data spanning the Neolithic to Bronze Age period in Southern Italy, limiting our understanding of the broader demographic trajectories of the Italian peninsula during antiquity. Genetic information is, in fact, largely unexplored for prehistoric Southern Italy, and just a handful of human palaeogenetic data are available for the territory of modern-day Apulia, Campania^17–21^ and Calabria^22,23^. To address this, we analysed palaeogenomic data from the scattered human remains recovered at Grotta della Monaca, one of the most extensively investigated cave sites in northwestern Calabria (Fig. 1a, b). Located over 600 metres above sea level, this karstic cave extends nearly 500 metres into a limestone ridge which is part of the Pollino massif in the Southern Apennines (Fig. 1c), approximately 20 km inland from the Tyrrhenian Sea. The innermost tunnels of the cavity were used as a burial ground during the Middle Bronze Age (MBA), as testified by the commingled remains attributed to secondary depositions found in area *m5v* (Fig. 1d). Radiocarbon dates were generated from 10 human bones collected in various areas of the cavity. The dates place the use of the funerary ground *m5v* between 1780 and 1380 cal. BCE (2σ). The remains in area *m5v* were found in close association with Protoapenninic pottery^24^, an archaeological facies well documented at coastal sites along the Adriatic and Ionian Southern Italy, and sporadically attested in the more mountainous territories of the Thyrrenian side. Beyond sector *m5v*, extensive and prolonged anthropic activity in post-medieval times led to the dispersal of numerous skeletal fragments, which were occasionally recovered in other parts of the cavity. Three isolated fragments found in areas *Ct.dx/fv* and *b* were radiocarbon dated to the same time range as the remains in area *m5v*. However, area *b* also yielded an isolated human bone fragment dated to the Early Eneolithic period (Supplementary Table 1). The scarcity and sporadic nature of these scattered elements, in contrast to the more coherent secondary depositions documented in area *m5v*, warrant caution when interpreting areas *Ct.dx/fv* and *b* – as well as other sectors where isolated human fragments were encountered – as formal funerary contexts. Moreover, the presence in these sectors of pottery clusters attributable to the Eneolithic Piano Conte and Laterza horizons suggests that other areas of the cave may also have been used as a burial ground during the Eneolithic. A detailed reconstruction of the archaeological context and anthropological material can be found in the Supplementary Information (Supplementary Notes 1 and 2). Overall, anthropological analysis established a minimum number of 24 individuals among the remains found in the cavity, with a greater frequency of infants aged between 0 and 12 years (62.5%). We sampled and processed for ancient DNA all available petrous bones and selected additional teeth only after visually assessing their preservation, specifically including only intact specimens that showed no signs of breakage or serious fracturing. According to our current knowledge, this study provides the first reconstruction of the genetic structure and demography of a Protoapennine community. We aim to fill critical gaps in ancient DNA data for the region and situate the findings within the broader context of prehistoric populations across Italy and the Mediterranean (Fig. 1e and Supplementary Data 1). By integrating genomic, archaeological, and anthropological evidence, the research provides insights into the prehistoric demographic history and the social dynamics of Middle Bronze Age communities in Calabria.

## Results

### Palaeogenomic dataset

DNA extracts were obtained from teeth (*n* = 10) and petrous bones (*n* = 13) and converted into multiple single-stranded non-uracil-DNA-glycosylase (UDG) treated libraries. All samples were sequenced after enriching for approximately 1,240,000 single-nucleotide polymorphisms (SNPs). For high-quality low-coverage samples reporting between 100,000 and 300,000 SNPs (GMO001, GMO009, GMO010, and GMO012), a second round of enrichment was performed on partial-UDG-treated libraries using the Twist capture protocol^25^ to increase SNP coverage and ensure robust performance in population genetic analyses. Palaeogenomic data were successfully generated for 14 individuals (see Table 1 and Supplementary Data 2). Nine ultra-low coverage samples (between 1000 and 10,000 SNPs) were only evaluated for sex and kinship relationships. Among the high-quality genomes (>100,000 SNPs), two were directly radiocarbon dated specifically for this study (GMO005 and GMO007), extending to 12 the number of direct dates available for the entire site (Supplementary Table 1 and Supplementary Note 1). Average fragment length and median rate of cytosine-to-thymine damage at the terminal nucleotides of the reads were consistent with the age of all samples, and no patterns of contamination were found at the nuclear or mitochondrial level for samples that passed the threshold for quality, except the male individual GMO009, for which we estimated 12% contamination on the X-chromosome (Supplementary Data 3-5). After retaining only post-mortem damaged (PMD) reads, we performed comparative principal component analysis (PCA) and excluded further contamination for the filtered sample GMO009, which was incorporated in subsequent steps (Supplementary Note 4 and Supplementary Fig. 3). For PCA, samples with less than 20,000 SNPs were filtered, whereas for subsequent population genetic analysis, individuals that were genetically related or of low coverage (<100,000 SNPs covered) were excluded.

**Table 1:** Summary statistics for the palaeogenomic dataset from Grotta della Monaca

Sample	Date	Genetic sex and anthropological age	Area	# SNPs on 1240 K	mtDNA (Hit quality)	Y-chromosome
GMO001	1690–1380 BCE (Indirect)	Adult female	m5v	1168765	K1a3 (85%)	(Female)
GMO002	3804–1380 BCE (Context)	Adult male	b	960	(Low coverage)	(Low coverage)
GMO003	3804–1380 BCE (Context)	Adult male	fv	3708	(Low coverage)	(Low coverage)
GMO004	1690–1380 BCE (Indirect)	Infant male	m5v	88704	(Low coverage)	G2a2b2a (PF3330)
GMO005	1616 BCE [95.4%] 1442 BCE (Direct)	Adult female	m5v	746979	H + 152 C! (95%)	(Female)
GMO006*	1690–1380 BCE (Indirect)	Infant male	m5v	690847	H5a + 152 C! (95%)	I2a1b2 (S2599)
GMO007	1692 BCE [90.4%] 1512 BCE (Direct)	Pre-adolescent male	m5v	621831	J1c1 (97%)	H2a1~ (Z18988)
GMO008	1690–1380 BCE (Context)	Pre-adolescent female	m5v	6378	(Low coverage)	(Female)
GMO009	1690–1380 (Indirect)	Male (age n/a)	CTsx.i	1085009	H3am (96%)	R1b1b (PF6333)
GMO010	1690–1380 (Indirect)	Female (age n/a)	S7	1130330	U8b1b1 (96%)	(Female)
GMO011	3804–1380 BCE (Context)	Female (age n/a)	S7	31522	U1a1a (97%)	(Female)
GMO012	1690–1380 (Indirect)	Female (age n/a)	S7	1165481	H2c (96%)	(Female)
GMO013	3804–1380 BCE (Context)	Infant female	fv	6589	T2c1 (72%)	(Female)
GMO014	3804–1380 BCE (Context)	Unknown (age n/a)	b	307	(Low coverage)	(Low coverage)
GMO015	1690–1380 (Indirect)	Infant male	S7	423265	H1e (96%)	R1b1a1b (PF6433)
GMO016	3804–1380 BCE (Context)	Unknown (age n/a)	S8	384	(Low coverage)	(Low coverage)
GMO017	3804–1380 BCE (Context)	Unknown (age n/a)	S4	3883	(Low coverage)	(Low coverage)
GMO018	1670 BCE [95.4%] 1430 BCE (Direct)	Adult female	m5v	760173	K1a2a (97%)	(Female)
GMO019	1560 BCE [92.1%] 1380 BCE (Direct)	Young female	m5v	7709	(Low coverage)	(Female)
GMO020	1690–1380 BCE (Context)	Infant female	m5v	1886	(Low coverage)	(Female)
GMO021	1690–1380 BCE (Indirect)	Infant male	m5v	330984	H5 + 152 C (91%)	I2a1b2 (Y10705)
GMO022	1690–1380 BCE (Indirect)	Adult male	m5v	204535	H1e (88%)	H2~ (SK1189)
GMO023	1690–1380 BCE (Indirect)	Infant male	m5v	12448	(Low coverage)	(Low coverage)

In the Date column, Direct refers to skeletal material directly radiocarbon dated; Indirect denotes samples dated by integrating genomic information with archaeological context; and Context indicates samples assigned chronologically based solely on the archaeological assessment of the finding area. Data for individual GMO006 are reported after merging same-source libraries GMO021 and GMO023. Mitochondrial haplogroup hit quality scores (in parenthesis) are reported from Haplogrep best-hit assignments.

### Community structure and parent-offspring union

**Fig. 2:**
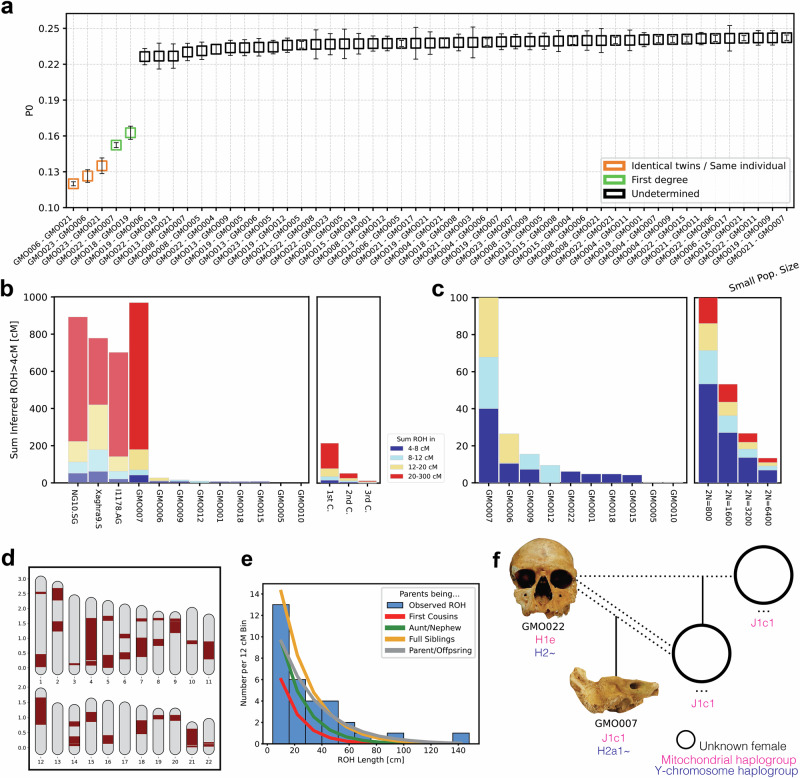
Genetic relatedness, inbreeding and pedigree reconstruction in the community from Grotta della Monaca. **a** Pairwise mismatch rate values among 20 individuals with at least 1000 SNPs. Each point represents a unique pairwise comparison between two individuals, and error bars indicate the standard error of the mean PMR across all overlapping SNPs for that pair; **b** Sum of runs of homozygosity (ROH) segments >4 cM calculated from the Grotta della Monaca community and a set of the most inbred individuals found in the literature. Colours separate ROH length classes in 4–8 cM (blue), 8–12 cM (sky blue), 12-20 cM (yellow) and >20 cM (red); expectations for close-kin unions is displayed on the right; **c** diffuse patterns of shorter ROH segments between the individuals from Grotta della Monaca, evidence of background relatedness. Expected ROH for small populations is shown on the right; **d** fractions of ROH segments in autosomal chromosomes and **e** histogram of >12 cM ROH length distribution for the extremely inbred individual GMO007; **f** the unusual pedigree reconstructed for individuals GMO022 and GMO007. Dotted lines denote reproductive union, while da ouble dotted line represents a consanguineous union. All published data are taken from the AADR repository v62, freely available at https://dataverse.harvard.edu/dataverse/reich_lab.

We combined genomic data and archaeological information to investigate the spatial organization of the burial ground. Based on pairwise mismatch rate calculation, three samples originate from the same individual (Methods, Fig. 2a and Supplementary Data 6). Hence, we merged these data into a single genetic profile, GMO006, yielding more than 690,000 SNPs. Genetic sexing identified ten females and eight males among the sparse skeletal material in the cavity. Due to low coverage, we could not reconstruct genetic sex for individuals GMO014 and GMO016, and attribution for individual GMO017 was considered unreliable. Notably, except for one adult male (GMO022), all individuals from burial area *m5v* (*n* = 10) were either adult females or juvenile-to-infant subjects. The analysis of uniparentally inherited markers displays a heterogeneous picture for the funerary ground (Supplementary Data 7, 8). Among the 13 individuals with sufficient coverage for assigning haplogroups, we identified 12 distinct mitochondrial haplotypes, with only the adult male GMO022 and the infant male GMO015 sharing identical haplotypes from the mitochondrial haplogroup H1e (private mutations A6779G, T16127C, T16224C and T16519C). In contrast, the Y-chromosome haplotypes were slightly more homogenous, with four different haplogroups reconstructed from the six male individuals analysed. Two of these (GMO009 and GMO015) belong to a sub-branch of haplogroup R1b, with private mutations leading to the R1b1b and R1b1a1b haplotypes, respectively, while individuals GMO007 and GMO022 share the H2~ Y-chromosome lineage (Supplementary Note 3).

Analysis of genetic relationships within the burial site identified two cases of first-degree relatives (Fig. 2a, Supplementary Data 9, 10). Using KIN^26^ we classified these relationships as parent-offspring pairs: intriguingly, both GMO007-GMO022 and GMO018-GMO019 originate from burial area *m5v*. Based on the estimated age at death of the skeletal remains, the adult GMO022 was determined to be the father of GMO007, a pre-adolescent male. In the case of the two female individuals GMO018 (likely 30+ years at death) and GMO019 (possibly 20+ years at death), the commingled nature of the skeletal remains and the absence of a shared relative prevented us from distinguishing which one was the mother and which the daughter. We assessed potential distant relationship ties using identity-by-descent (IBD) segments inferred by ancIBD^27^ on individuals with over 600,000 genotyped SNPs. The majority of the individuals did not exhibit evidence of distant genetic relatedness, except for one case: the adult female individual GMO005 and the juvenile male GMO006, both found in burial sector *m5v*, shared 10 long IBD segments ( > 12 cM), indicative of a fourth-degree relationship (Supplementary Data 11).

To test the possibility of inbreeding events within the community of Grotta della Monaca, we measured runs of homozygosity (ROH) segments in 10 individuals with sufficient genomic coverage (>400,000 SNPs) using hapROH^28^ (Fig. 2b). Briefly, an abundance of ROH segments >20 centimorgans (cM) long corresponds to consanguineous mating of an individual’s parents, while an increased number of short runs indicates distant parental relatedness, which is commonly observed in small or isolated populations. Only two of the analysed individuals (GMO006 and GMO009) carried several short ROH longer than 4 cM (Fig. 2c, Supplementary Data 12), summing up to a total ROH length of 26 cM and 18 cM, respectively. These results indicate that the parents of the two individuals were distantly related, possibly pointing to the last 6–10 generations. Additionally, four individuals exhibit few short ROH, no longer than 5 cM, suggesting a diffused pattern of remote inbreeding in the community. Based on the inferred ROH, we estimated the effective population size (Ne) of our samples and compared it to a subset of relevant Bronze Age populations taken from the literature. Our estimates from the community of Grotta della Monaca corresponded to close to 5000 reproducing individuals (Ne = 4698, 2610–9778 95% CI, Supplementary Data 13), similar to the population size calculated from MBA communities in Iberia (Ne = 3167, 1470–8814 95% CI) and Croatia (Ne = 3018, 1564–7016 95% CI), but remarkably smaller than of groups from the coeval Tumulus culture in Central Europe (Ne=10624, 3441-50000 95% CI), or the large Middle to Late Bronze Age urban centre of Alalakh in Northern Levant (Ne = 79537, 18,123–50,0000 95% CI). Notably, individual GMO007 exhibits the highest sum of long ROH segments ever reported in ancient genomic datasets to date (Fig. 2b). Nearly 800 cM of his genome consists of ROH segments longer than 20 cM, with the longest stretch extending 142 cM. Such segments were distributed across large fractions of the autosomal chromosomes (Figs. 2d and e), providing indisputable evidence that the young male was the offspring of a first-degree incestuous union. This result stands as an exceptionally rare and remarkable finding, the earliest identified in the archaeological record, with only three comparable instances among ancient populations to date. One extremely inbred individual has been reported in Middle Neolithic Ireland^29^ (NG10.SG), radiocarbon dated to 3339–3028 cal. BCE, which showed nearly 670 cM of >20 cM ROH segments, the longest reaching 65 cM. Another individual from Chalcolithic Israel^30^ (I1178.AG), archaeologically dated to 4500–3500 BCE, reported 560 cM of ROH longer than 20 cM and the longest stretch reaching 91 cM. A third individual from Late Neolithic Malta^31^ (Xaghra9.SG), radiocarbon dated to 2530-2400 cal. BCE, exhibited a total of approximately 360 cM of >20 cM ROH segments, including one stretch measuring 111 cM (Supplementary Data 12).

Through kinship analysis, we identified the father of GMO007 being GMO022, but not the mother. Leveraging uniparentally inherited genetic markers, we find that the two individuals do not share a matrilineal connection. This genetic evidence unambiguously reveals that GMO007 was the son of GMO022 and his daughter (Fig. 2f).

### The genomic makeup of the Middle Bronze Age community from Grotta della Monaca

**Fig. 3:**
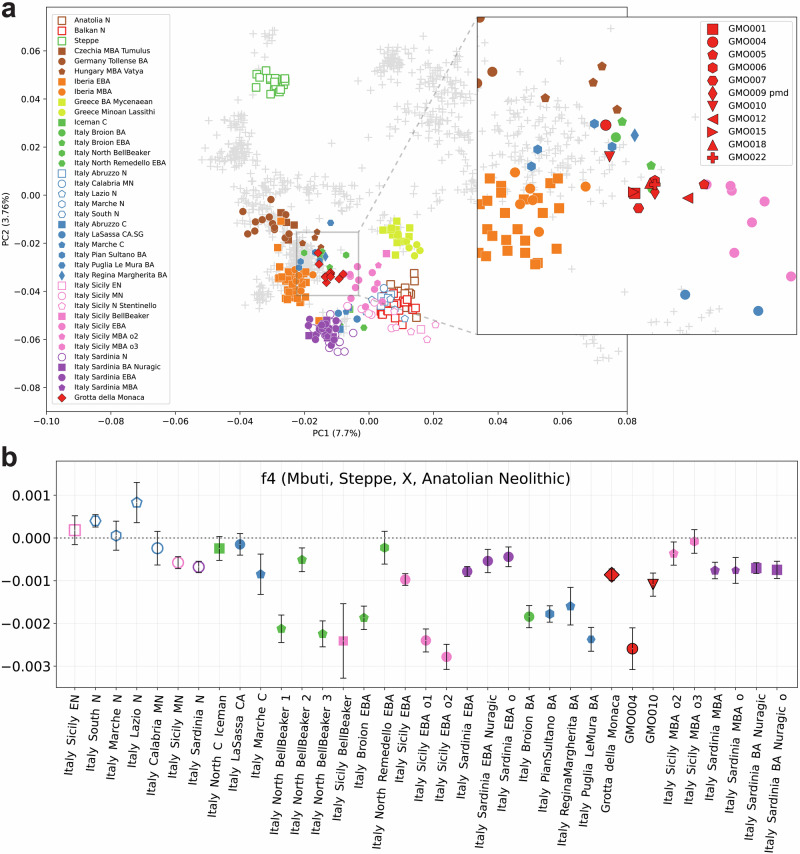
Population structure and Steppe-related ancestry in Bronze Age Italians. **a** Principal component analysis calculated on a set of present-day western Eurasians (n = 1074) from the Human Origin Affymetrix dataset. Projected ancient individuals (n = 278) are displayed as coloured shapes; the zoom in shows GMO individuals (red) forming two separate clusters; **b** genetic variation in Steppe-related ancestry among Italian individuals from the Neolithic to the Middle Bronze Age assessed using f₄-statistics. Each point represents one f₄-statistic per population; error bars indicate the standard error of each statistic, calculated as f₄ divided by the corresponding Z-score. Populations are ordered chronologically from left to right. Symbol shapes correspond to population groupings as shown in the legend of Fig. 3a. All published data are taken from the AADR repository v62, freely available at https://dataverse.harvard.edu/dataverse/reich_lab.

We explored the biogeographical origin and ancestral structure of the MBA community from Grotta della Monaca by performing a principal component analysis, projecting the newly generated and relevant published ancient DNA data onto the genetic variation calculated from 1074 present-day western Eurasians (Supplementary Data 14). Most of the individuals from Grotta della Monaca form a distinct, relatively homogeneous cluster located in the PCA space between Bronze Age Central Europeans, Early (EBA) and MBA Iberians, and Bronze Age (BA) Sicilians (Fig. 3a). We observe the closest genetic similarity to published data from the Italian peninsula, specifically to EBA and MBA individuals from the Northeast (Italy Broion EBA/MBA) and Sicily (Italy Sicily EBA), suggesting a high degree of genetic continuity within these regions during this period. A modest shift towards coeval populations from Central Europe is visible for two individuals (GMO004 and GMO010), suggesting an increased genetic affinity with individuals from EBA and MBA North-Central Italy (Italy North Bell Beaker and Italy Pian Sultano BA) and Hungary (Hungary MBA Vatya). Moreover, two individuals (GMO005 and GMO012) exhibit a slight shift towards Early Bronze Age Sicilians; however, formal statistical tests do not indicate significant genetic heterogeneity between these individuals and the main cluster (Supplementary Data 15).

The genetic attraction towards Bronze Age Central Europeans observed in some of our individuals may reflect different proportions of ancestry associated with Western Steppe pastoralists, who made a large contribution in shaping the genomic make-up of Bronze Age Eurasian populations. To determine whether the patterns observed in PCA reflect genuine differences in genetic ancestry, we assessed pairwise cladality between individuals using qpWave^32,33^. While cladality could not be rejected between individuals from Grotta della Monaca and Early Bronze Age Sicilians, a single individual, GMO010, showed signals of rejection of the proposed model at *p* < 0.01 when compared to almost all other individuals from the same community (*p*-values ranging 1.29e–06 to 0.0126, Supplementary Note 5, Supplementary Fig. 4 and Supplementary Data 16). This outlier reported affinity only with individual GMO004 (*p* = 0.281), matching the observed clustering in the PCA, and exhibited increased attraction to Early and Middle Bronze Age groups from Northern Italy. We classified individuals GMO004 and GMO010 as genetic outliers for further group-based analyses, but marked results for individual GMO004 as statistically underpowered due to low coverage (<100,000 SNPs).

We then assessed variation in Steppe-related ancestry among prehistoric individuals from Italy – including the newly reported GMO group – by running f-statistics in the form ƒ4(Mbuti, Steppe; X, Anatolia N), where X represents prehistoric Italian individuals. The Steppe and Anatolia N groups served as proxies for the BA Western Steppe pastoralists and the Early Neolithic Farmers from Anatolia, respectively. Consistent with previous findings, published EBA and MBA individuals from Northern, Central, and Southern Italy exhibit affinity to Steppe-derived ancestry (Fig. 3b and Supplementary Data 17). This result is in line with existing evidence for the presence of this genetic component in prehistoric Italian populations by at least 2300 BCE in the mainland^34^ and by 2400 BCE in Sicily^33^. Here, this pattern is also clearly visible in the Grotta della Monaca cluster (f4 = −0.0008, Z = −6.332), and extremely evident for GMO004 (f4 = −0.0025, Z = −5,307), while no remarkable differences are present for GMO010 (f4 = −0.0011, Z = -4.035).

**Fig. 4:**
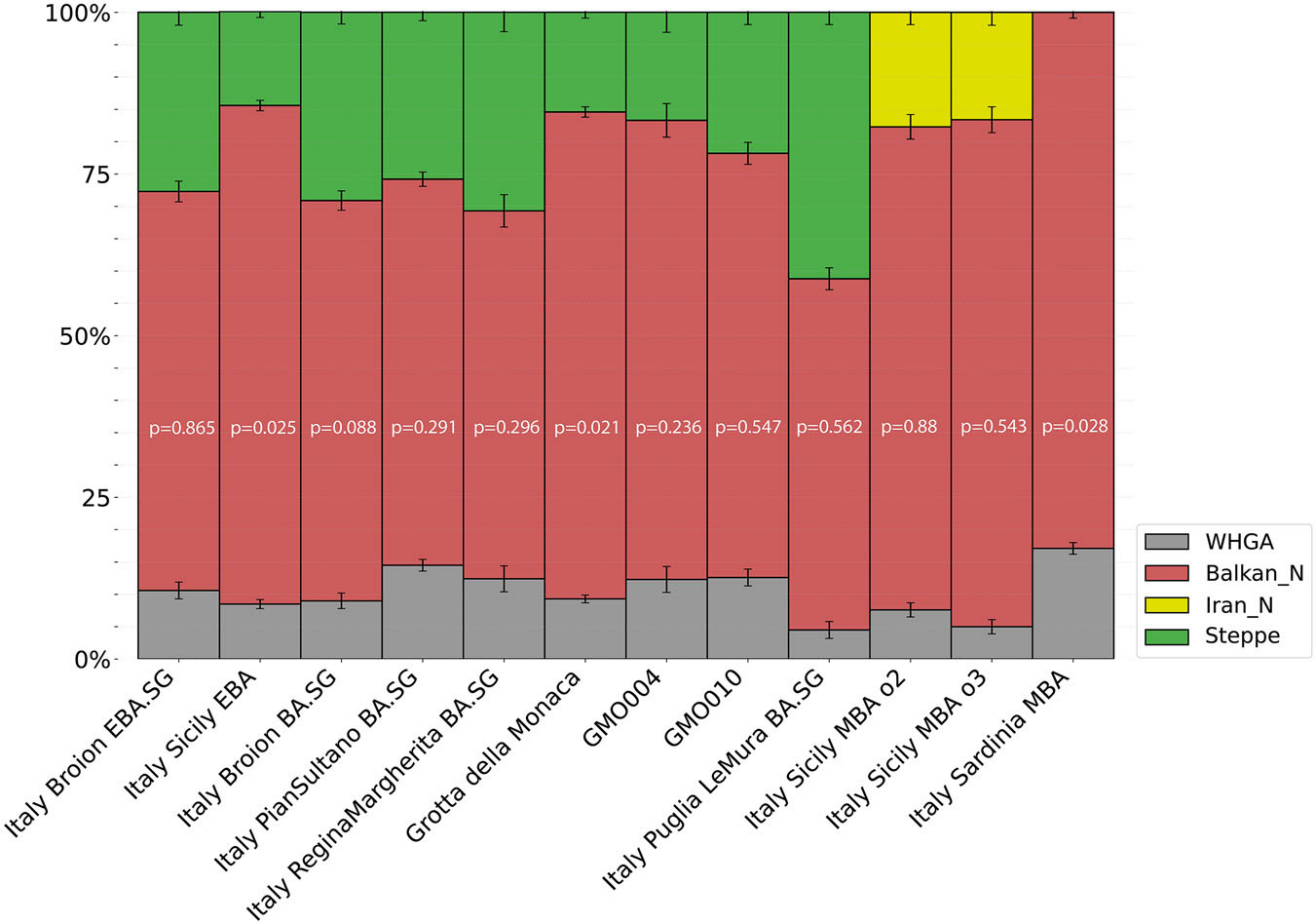
qpAdm ancestry modelling of Grotta della Monaca individuals and coeval Italian populations. Each bar represents the ancestry proportions inferred from a single qpAdm model for a population, using a three-way distal approach. Error bars indicate the standard errors of the ancestry coefficients as estimated by qpAdm. All published data are taken from the AADR repository v62, freely available at https://dataverse.harvard.edu/dataverse/reich_lab.

Subsequently, we modelled the genomic makeup of individuals from Grotta della Monaca as a combination of three ancestral proxies representing the three major contributors to European populations from the 3rd millennium BCE to the present day^35^, i.e., Western hunter-gatherers (WHG), the early European farmers (EEF), and the aforementioned western Steppe herders (Supplementary Data 18). We first tested the samples individually to support our clustering results (Supplementary Data 19). Then, using a distal approach for admixture modelling^36^ (Supplementary Note 6), we estimated that the GMO group derives 75.3% ± 0.8% of EEF ancestry when using Balkan N as source of EEF ancestry, 15.4% ± 0.9% ancestry from the Steppe, and 9.3% ± 0.6% from a subset of late Epigravettian and Mesolithic Hunter-Gatherers from Western Europe (WHGA) (Fig. 4). We estimated analogous proportions of hunter-gatherer ancestry in previously reported Italian Bronze Age groups, including Italy Broion BA (9% ± 1.2%), Italy North Bell Beaker 3 (9.4% ± 1.4%) and Italy Sicily EBA (8.5% ± 0.7%). Notably, the observed proportions of EEF and Steppe-related ancestries of the GMO cluster closely align with those of Early Bronze Age Sicilians (77.1% ± 0.8% and 14.5% ± 0.9%, respectively). The proportion of Steppe ancestry marks the most evident difference between the GMO cluster and the outlier GMO010. The latter exhibits, compared to the main genetic cluster, a higher affinity with Steppe populations (21.8% ± 1.9% Steppe-derived ancestry), a slightly greater proportion of hunter-gatherer ancestry (12.6% ± 1.3%), and a lower proportion of Neolithic farmer ancestry (65.6% ± 1.7%) (Supplementary Data 20). These patterns reinforce the PCA results of this individual and are shared with the second outlier GMO004, which however, we caution was slightly below the suggested cutoff for admixture modelling (~80,000 SNPs).

Motivated by previous evidence of Eastern-like ancestry in MBA Sicily^33^, we tested whether the Grotta della Monaca population carried genetic contributions from sources such as Caucasus Hunter-Gatherers (CHG) or Neolithic Iranian farmers, which have been used as proxies for Levantine-related ancestry. However, our qpAdm models did not provide a statistically feasible fit for either the GMO cluster or the genetic outlier GMO010. Similarly, we tested for North African ancestry, previously reported in Bronze Age and Punic-period samples from Sardinia^33,37^, but found no evidence of such genetic contributions in the Grotta della Monaca individuals.

To further explore possible sources of local ancestry, we tested several scenarios employing a proximal modelling approach (Supplementary Data 21). We leveraged the limited data available for Neolithic individuals from the Italian peninsula and surrounding islands to assess local farmer ancestry. Additionally, we incorporated Early Bronze Age individuals from either Northern Italy or Sicily to trace for a local origin of Steppe ancestry components. Regarding the outlier GMO010, models requiring the Grotta della Monaca cluster as a single source of ancestry did not fit its genomic composition. Instead, its ancestry was best explained by a model deriving the entire ancestry from the Early Bronze Age northeastern Italian group of Broion (*p* = 0.26), which confirms genetic affinities observed in previous results. Conversely, no-single source models successfully explained the genetic makeup of the main GMO cluster. In fact, robust two-way models required – alongside northern Italian EBA ancestry – a Neolithic or Middle Neolithic contribution ranging 30.2% ± 3.7% to 46.8% ± 4.9%, sourced from individuals in Central Italy (Italy Lazio N, *p* = 0.37), Southern Italy (Italy Calabria MN, *p* = 0.03) or Sicily (Italy Sicily MN, *p* = 0.11 and Italy Sicily Stentinello, *p* = 0.06).

Finally, we examined a panel of SNPs associated with functional and phenotypic traits (Supplementary Data 22). First, we explored 41 informative SNPs from the HirisPlex-S DNA test system^38^, and found alleles for blue eyes in six out of nine individuals with sufficient genomic coverage (Supplementary Data 23). Hair colour analysis revealed that almost all the individuals exhibited dark hair, either brown (*n* = 5) or red (*n* = 6). Additionally, the majority of the individuals reported general darker skin pigmentation, except for individuals GMO001 (*p* = 0.98, AUC loss = 0.001), GMO015 (*p* = 0.92, AUC loss = 0.05), GMO018 (*p* = 0.45–0.49, AUC loss = 0.02), and GMO022 (*p* = 0.55, AUC loss=0.04) who carried alleles linked to pale skin. Individuals GMO018 and GMO022 also carried genetic variants associated with lighter hair coloration (*p* = 0.86 and *p* = 0.89, AUC loss = 0.002 and 0, respectively).

We also explored functional SNPs annotated in the SNPedia and dbSNP datasets. We found that all individuals with sufficient genomic coverage carried ancestral alleles at SNPs associated with lactase persistence, suggesting that they might have suffered from lactose intolerance in adulthood. Given the observed inbreeding events within the population, we investigated SNPs associated with rare diseases to assess potential health implications. However, no notable genetic markers associated with inherited diseases were identified. One exception is the female individual GMO012, who carried the derived allele on the SNP rs8063 located on the gene *UBE2I*. This variant has been significantly associated with mild cognitive impairment in women, even though its phenotypic impact in this individual remains uncertain.

## Discussion

The occupational and demographic patterns of Protoapennine populations in Southern Italy are characterized by significant contrasts between the more densely inhabited eastern regions and the more sporadically occupied western ones, shaped by a combination of geographical constraints and socio-cultural dynamics. In Apulia, Basilicata, and Ionian Calabria, the archaeological record primarily points to medium-sized sites located on plains or coastal areas. These were largely integrated into cultural and trade networks with the Italian mainland and Adriatic maritime routes. By contrast, Protoapennine occupation in the southern Tyrrhenian regions is defined by sporadic and isolated promontory sites, which exhibit closer affinities with Tyrrhenian Central Italy, the Aeolian Islands, and northeastern Sicily. Our results show that the individuals from Grotta della Monaca display a strong affinity with Early Bronze Age Sicilians, exhibiting similar proportions of hunter-gatherers, Anatolian farmers, and steppe herders ancestry. In particular, steppe ancestry emerges as the major genomic distinction between this Middle Bronze Age community in Calabria and coeval individuals from the Italian peninsula.

By 1700 BCE ca., Aegean-type pottery appears in the Aeolian islands, Sicily, and in cave contexts associated with Protoapennine groups in western Calabria cave, reflecting the influence of early contacts with sea peoples of Aegean origin^39–41^. In western Sicily, such interactions coincided with the emergence of eastern Mediterranean ancestry around 1800-1500 BCE. By the same period, our results highlight that this ancestry is absent from the genomic make-up of the individuals at Grotta della Monaca.

The limited resources and the hardness of territories in Tyrrhenian Calabria led to the diffusion of small, dispersed settlements in mountain areas^14^. These may have possibly clustered together in short-lived communities, culminating in the emergence of polycentric systems that remained relatively primitive in terms of socio-economic development when compared to coastal and lowland areas of the mainland and Sicily^42^. Interestingly, genomic data reveal that several individuals carried ancestral alleles at SNPs associated with lactase persistence, indicating a likely susceptibility to lactose intolerance in adulthood. This finding, when considered alongside isotopic evidence for the consumption of dairy and meat products at the site^43^, suggests that members of this community consumed milk and dairy products despite their genetic intolerance. This apparent mismatch between dietary practice and genetic predisposition may further reflect a context of limited dietary choices and a subsistence economy mostly reliant on pastoral resources. Within this scenario, the use of caves as communal cemeteries or cultic spaces has been suggested to be a pivotal sign in shaping the identity of the community’s sense of self. Yet, the practice of using natural cavities for funerary purposes during the MBA remains a topic of active debate in Central and Southern Italy, and the organizational structure of Protoapennine societies is still uncertain. Collective inhumations associated with Protoapennine groups typically took place in enclosed spaces of either natural or artificial origin. The best-known examples include Grotta Vittorio Vecchi and Grotta Regina Margherita in Lazio, Grotta Manacora in Apulia, and Grotta della Monaca in Calabria; other instances also include dolmens and artificial hypogea. While some scholars proposed that these sites primarily served as worship areas for residential groups^44^, others suggest a strict tie to kinship expressions within a single, distinct community^45^.

At Grotta della Monaca, the absence of a nearby settlement and a lack of clear mortuary differentiation prompted us to rely primarily on biological data to interpret social organization. Our findings suggest that the commingled remains found in sector *m5v* belong to the members of a specific Protoapennine group, for which a consistent number of radiocarbon dates (eight for this sector, see Supplementary Table 1) indicate they lived around 1780–1380 BCE. Although anthropic disturbance, diagenesis, and sampling strategy limited the reconstruction of an extended pedigree, our genetic results support a homogeneous community structure. These include a clustered genetic profile, diffused patterns of consanguinity, and possible sex- and age-biased burial practices in sector *m5v*. However, given the presence of scattered remains in other sectors of the cave, one of which is dated to the Early Eneolithic period, we caution that this latter interpretation requires further investigation of the archaeo-anthropological material still present in the cave. Despite recurrent patterns of background relatedness, our estimates do not suggest a highly isolated or extremely small population, aligning instead with archaeological theories that emphasize interconnectivity between communities in the mountainous areas of northwestern Calabria. This is also confirmed by the presence of at least two outliers in the group, which exhibit a higher affinity to Early Bronze Age northeastern Italian populations. The observed genetic heterogeneity within the burial group likely reflects a degree of interconnectivity along the Italian peninsula. Overall, our results point toward differentiated population histories within the Middle Bronze Age group of Grotta della Monaca, suggesting that interregional mobility and interactions may have played a role in shaping the local genetic diversity of Protoapennine community members.

One particularly rare finding is the extreme case of inbreeding highlighted in one of the pedigrees - a unicum in the archaeological record to date. Archaeogenetic research has linked close-kin mating events to isolation and small population size among different human groups, including Neanderthals^46^, Upper Palaeolithic hunter-gatherers^47,48^, and Neolithic agriculturalists from insular contexts^31^; others connected inbreeding to dynastic or royal systems in settled communities, suggesting social strategies for intensifying hierarchy and consolidating territorial power in ancient societies^29,49–51^. These scenarios mostly involve full or half-sibling mating, and have also been associated with megalithic architecture and public rituals. Our results prove instead that such unusual behaviours can refer to different social contexts, reflecting a variety of cultural aspects not necessarily involving elite communities or extremely small populations, as also recently suggested^52^. Hence, interpreting extreme evidence such as the reproductive union between parent and offspring requires a critical evaluation of the entire archaeo-anthropological context.

The reproductive union between parent and offspring observed in our study may reflect a socially sanctioned behaviour, as proposed by some authors^29^. This might potentially explain the presence of individual GMO022 as the only male adult buried in the female- and non-adult-dominated funerary ground *m5v*. However, the chaotic dispersal of the remains and the unclear organization of the burial area demand careful consideration in drawing definitive conclusions. It remains uncertain whether this event represented a) a tolerated marital custom within Protoapennine groups, hence an exception to typical mating patterns; b) a singular transgression; or c) the result of coercion or violence. Anthropological literature suggests that the interpretation of incestuous events as taboo is primarily rooted in a spontaneous human aversion to the act itself, rather than to its biological consequences, since, when non-violent, the act may in fact be inherently harmless^53^. This calls for a cautious approach in interpretation.

Our study represents an important step forward in providing evidence for understanding cultural practices of prehistoric communities, despite the interpretive challenges posed by such an extreme case. By leveraging the constantly growing potential of archaeogenetics analysis, we have reconstructed the demographic profile of a small Protoapennine community and contributed unprecedented perspectives on demography, social organization, and cultural expression in Bronze Age Southern Italy. Our findings show that atypical biological patterns can correspond to a range of social practices, emphasizing the necessity of further archaeological, anthropological and genetic investigations to clarify the complexity of kinship systems and social practices in prehistoric Southern Italy.

## Methods

### AMS C14 dating

During more than 20 years of investigations, multiple samples taken from Grotta della Monaca were submitted for radiocarbon analysis. Two samples, not associated with any known individual, were radiocarbon dated in 2004 at the University of Groningen. Subsequent analyses by means of accelerator mass spectrometry were performed in 2008, 2022 and 2024 at the Centre for Applied Physics, Dating, and Diagnostics (CEDAD), Department of Mathematics and Physics at the University of Salento. For the two individuals (GMO005 and GMO007) specifically dated for this study after palaeogenetic analysis, collagen extraction from the samples was performed using the Longin method^54^ at the chemical laboratories of the CEDAD, following internal procedures^55^. The selected collagen fraction was converted into CO₂ by combustion in sealed quartz tubes containing CuO and silver wool. The CO₂ was then reduced to graphite at 600 °C using hydrogen gas and iron powder as a catalyst. The AMS radiocarbon analyses were conducted using a 3 MV Tandetron accelerator (High Voltage Engineering Europa BV, model 413oHC) following established procedures^56^. The obtained ¹⁴C/¹²C ratios were corrected for isotopic fractionation based on δ¹³C values measured directly by the AMS system, as well as for background contributions from both the instrument and the chemical preparation. Conventional radiocarbon ages were computed following the method by Stuiver and Polach^57^, and calibrated using the latest IntCal20 calibration curve. With the single exception of a scattered bone fragment found in sector *b* of the cavity, and dated to 3804-3643 cal. BCE, all the radiocarbon dates fall in the range 1780-1380 cal. BCE (Supplementary Table 1 and Supplementary Note 1), coherently pointing to the utilization of the necropolis during the Southern Italian Middle Bronze Age.

### aDNA data generation

The Soprintendenza Archeologia, Belle Arti e Paesaggio for the Catanzaro, Crotone and Cosenza provinces granted regulatory approval for the study and sampling of the anthropological material presented in this study (protocol number 0015731, 24/12/2018). Sample selection first focused on the commingled remains of the area *m5v*, from which we selected eight left and three right petrous bones, along with one deciduous molar, the latter associated with known infant 7. Given the absence of pars petrosa among the scattered remains found sparse in other sectors of the cavity, we sampled teeth and relied on genetic analysis for individual discrimination.

Preliminary screening of samples for endogenous DNA content was conducted in the Ancient DNA facilities of the Department of Cultural Heritage (University of Bologna) following standard procedure for aDNA analysis from petrous bones and teeth^58–60^; an extraction-to-indexing approach was later performed at the Pharmapark laboratory in Jena following internal procedures based on standard palaeogenetic protocols^61–63^. The 1240 K SNPs capture enrichment was performed for 23 samples, and the libraries were sequenced at ~20 million or ~60 million reads on 75 bp SE HiSeq 4000 System. Additional Twist capture^25^ was performed for six lower endogenous samples, which were sequenced on a 100 bp PE Novaseq X plus lane. A comparative PCA was later performed to test the co-analysability between 1240 K SNPs and Twist-enriched data (Supplementary Note 4).

Data processing was performed through EAGER v2.5.1^64^: Illumina adapters were removed from the sequences using AdapterRemoval v.2.2.0^65^ with a minimum adapter overlap ~1; reads from all the libraries were then aligned to the Full 1000genomes phase2 Reference Genome Sequence (hs37d5) using BWA version 0.7.12^66^, with minimum read quality ~20, length filters ~30, allowed mismatches threshold ~0.01, maximum edit distance ~2, and seed length ~1024. Mapping quality filtering was enabled and reads with a quality score <30 were excluded from the resulting BAM files. PCR duplicates were removed using MarkDuplicates from Picard^67^. Damage patterns were calculated from BAM files with DamageProfiler^68^ using a length filter ~100 and a base threshold ~30 for inclusion in calculations of deamination frequency. The *run_trim_bam* option was used to clip 10 bases from both the left and right ends of single-stranded which did not undergo UDG treatment using the R package bamUtils^69^. Genotyping was performed using the pseudohaploid-specific genotype caller Pileupcaller^70^ using as reference sites the complete list of the 1,24 million SNPs involved in the enrichment step. Sex estimation was performed using Sex.DetERRmine^71^, while nuclear contamination was initially estimated using the ANGSD method for X chromosomes^72^. Report statistics were automatically generated by EAGER using QualiMap2^73^ and samtools^74^ and summarized with MultiQC^75^. The data was further authenticated by independently running a series of tools for contamination estimates, including ContamMix^76^, AuthentiCT^77^, and hapCon^78^. Samples that reported evidence of possible contamination in any of the estimates were further filtered to retain only post-mortem damaged (PMD) reads. We then performed a PCA of modern individuals and projected PMD and non-PMD filtered data to investigate whether shifts in genetic compositions could be explained by matters of contamination (Supplementary Note 4).

### Uniparentally inherited markers and kinship analysis

Statistical inference of genotype mismatches was used to discriminate individual genetic data from commingled remains as either originating from a single or multiple individuals. We first calculated the pairwise mismatch rate (P0) as in Kennett et al. 2017 and found an average mismatch rate of 0.24. The isolated left petrous bone GMO006 was found sharing a P0 of 0.124 (Z = 75) and 0.13 (Z = 14) with the isolated right petrous bone from known infant 23-GMO021 and a first upper left deciduous molar from known infant 7-GMO023, respectively. Hence, we merged data originating from the same source and replicated PMR calculations. Data were compared with the results obtained using the genotype likelihood estimators tools READv2^79^ and KIN^26^, which uses IBD segments to calculate kinship models and integrates a model that considers the possibility of inbreeding and contamination. Mitochondrial consensus sequences generated from ContamMix were used as input in Haplogrep3^80^ to obtain confident mitochondrial haplogroup classification. Y-chromosome haplogroup assignment was performed by investigating the presence of variants reported in the extended dataset ISOGG^81^. A specific Y-chromosome haplogroup was assigned when diagnostic sites were found, following the root-to-end evaluation of such SNPs presented in other publications^82–84^. ROH segments and population size were computed from pseudo-haploid genotypes using hapROH^28^ on samples with at least 400,000 SNPs overlapping the 1240 K SNPs dataset. The newly generated samples were analysed along with a subset of individuals from a relevant archaeological context that broadly matched the temporal span of our samples. For population size, we excluded inbred ( > 50 cM homozygous stretches) and closely related individuals. We ran ancIBD^27^ on a subset of prehistoric samples available from the literature and report the only pair involving GMOs with at least one shared copy of 20 cM length and *gp_fraction* > 0.7.

### Population genetic analysis

We co-analysed our data with two extensive genomic datasets obtained by sub-setting the publicly available genomic data from the AADR repository^85^ v62 provided by the David Reich Lab of the Harvard Medical School. We used smartpca from the EIGENSOFT package^86,87^ to compute principal components from a subset of 1074 present-day Western Eurasians (Supplementary Data 14) from 597,573 SNPs in the Human Origin dataset^32,35^ and projected relevant ancient individuals using the *lsqproject* option. The first two principal components resulting from the outputted eigenvectors were plotted using a custom script in Python.

ADMIXTOOLS v.4.1^32^ was used to compute *f*4-statistics using qpDstat. Mbuti.DG was set as the outgroup, and the statistics were computed from the same dataset in the form *f*4(Mbuti.DG, B; C, D), where B was the test population (or individual) and C/D pairs of populations (or individuals) that were tested for sharing alleles at an equal rate with B (Supplementary Data 15). A pairwise approach, similar to what was presented elsewhere^33^, was later employed using qpWave to test whether each individual from Grotta della Monaca was consistent with being from the same group as others from the same time period and geographical area, retaining only individuals with >100 K SNPs (Supplementary Note 5). We finally used qpAdm^36^ to estimate ancestral components modelling the genetic pool of the Grotta della Monaca population. Various tests were run, first using distantly related populations and then using a proximal modelling to test hypothetical sources of admixture more closely related in space and time to our population (Supplementary Note 6). The HirisPlex system^38^ was employed to infer hair, eye, and skin pigmentation for the individuals. Ancestral and derived alleles were identified at positions overlapping the 1240 K SNPs dataset and a curated subset of functional SNPs from SNPedia and dbSNP. These were then used to assess potential phenotypic traits and monogenic diseases, focusing on good-coverage ( ≥ 6x) transversion-only sites. To reduce deamination-induced biases, we performed preliminary processing of non-UDG-treated libraries by trimming 5′ ends at the extremities. The resulting filtered data are reported in Supplementary Data 22-23.

### Statistics and reproducibility

All statistical analyses were performed using established, open-access tools widely applied in archaeogenetic research, including smartpca (EIGENSOFT v6.1.4) for principal component analysis, qpWave and qpAdm (ADMIXTOOLS v4.1) for ancestry modelling, and ancIBD and hapROH for kinship and runs of homozygosity analyses. Statistical uncertainty for admixture proportions was estimated using the standard error output from qpAdm. Sample sizes correspond to the number of successfully genotyped individuals from Grotta della Monaca, as reported in Supplementary Tables and Notes. For ancient DNA data generation, each sample was processed as an independent biological replicate; for low-coverage individuals, sequencing libraries were prepared and enriched in duplicate to verify data consistency. All published comparative datasets were obtained from the AADR v62 public repository. Analyses were conducted using version-controlled pipelines (EAGER v2.5.1, ADMIXTOOLS v4.1, and in-house Python scripts), and all code and parameters used are available upon request to ensure reproducibility.

### Reporting summary

Further information on research design is available in the Nature Portfolio Reporting Summary linked to this article.

## Supplementary information

Supplementary Information

Description of Additional Supplementary Materials

Supplementary Data 1-23

Reporting Summary

## Data Availability

The raw sequencing data (FASTQ files) generated in this study are publicly available in the European Nucleotide Archive (ENA: https://www.ebi.ac.uk/ena/browser/home) under project accession number PRJEB94318. The AADR dataset^85^ is freely available at Harvard Dataverse (https://dataverse.harvard.edu/dataverse/reich_lab), and was used to generate Fig. 1e. The osteological remains are under the protection of the Soprintendenza Archeologia, Belle Arti e Paesaggio of the Cosenza province, and are formally deposited and catalogued at the municipal deposits in Sant’Agata d’Esaro (Protocol number 11710-P, 5/12/2023. Box identifiers A013, A033, A066, B009, B031, B035, B063, B093, B102, and B107). Source data underlying main figures are provided in Supplementary Data 6 (Fig. 2a), Supplementary Data 12 (Fig. 2b), Supplementary Data 13 (Fig. 2c), Supplementary Data 14 (Fig. 3a), Supplementary Data 17 (Fig. 3b), Supplementary Data 20 (Fig. 4).
