# Supplementary material for: Archaeogenetics reconstructs demography and extreme parental consanguinity in a Bronze Age community from Southern Italy
Source: Commun Biol. 2025 Dec 15;8:1766. doi: 10.1038/s42003-025-09194-2 (PMC12706022; doi:10.1038/s42003-025-09194-2)
Supplement: Supplementary file 1 — Supplementary Information [file 42003_2025_9194_MOESM1_ESM.pdf]

Supplementary Information

Archaeogenetics reconstructs demography and extreme parental consanguinity in a Bronze Age community from Southern Italy

List of contents

Supplementary Notes 2

Supplementary Note 1: Archaeological context 2

Supplementary Figure 1 3

Supplementary Table 1 4

Supplementary Figure 2 6

Supplementary Note 2: Anthropological remains 8

Supplementary Note 3: Uniparentally inherited haplogroups 13

Supplementary Note 4: PCA for capture and PMD strategy assessment 15

Supplementary Figure 3 17

Supplementary Table 2 18

Supplementary Table 3 18

Supplementary Note 5: Clustering individuals via pairwise qpWave 19

Supplementary Figure 4 20

Supplementary Note 6: qpAdm modelling 20

Supplementary References 22

Supplementary Notes

Supplementary Note 1: Archaeological context

(Francesco Fontani, Rossella Veneziano and Felice Larocca)

Grotta della Monaca (Sant'Agata di Esaro, Cosenza, Supplementary Fig. 1a) is a vast karst cavity located at 600 meters above sea level. It extends for almost 500 meters inside a limestone massif overlooking the upper valley of the Esaro River, in the territory between the Tyrrhenian Sea to the west and a vast alluvial plain to the east (Supplementary Fig. 1b). Mostly frequented by humans since prehistoric times because of the abundance of mineral deposits, over the course of twelve years archaeological investigations have been pursued by researchers from the University of Bari and the Regional Center of Speleology "Enzo dei Medici", headed by Dr. Felice Larocca. The cave is characterized by three separate sectors: the first is represented by an entrance duct, known as "Pregrotta" (Supplementary Fig. 1c); from the area of "Pregrotta" opens a central room, the "Sala dei Pipistrelli", a vast underground environment that extends for over 60 meters in length and 30 meters in width (Supplementary Fig. 1d). The inclined course of the room ends at the bottom in a flat area mostly consisting of clay deposit.

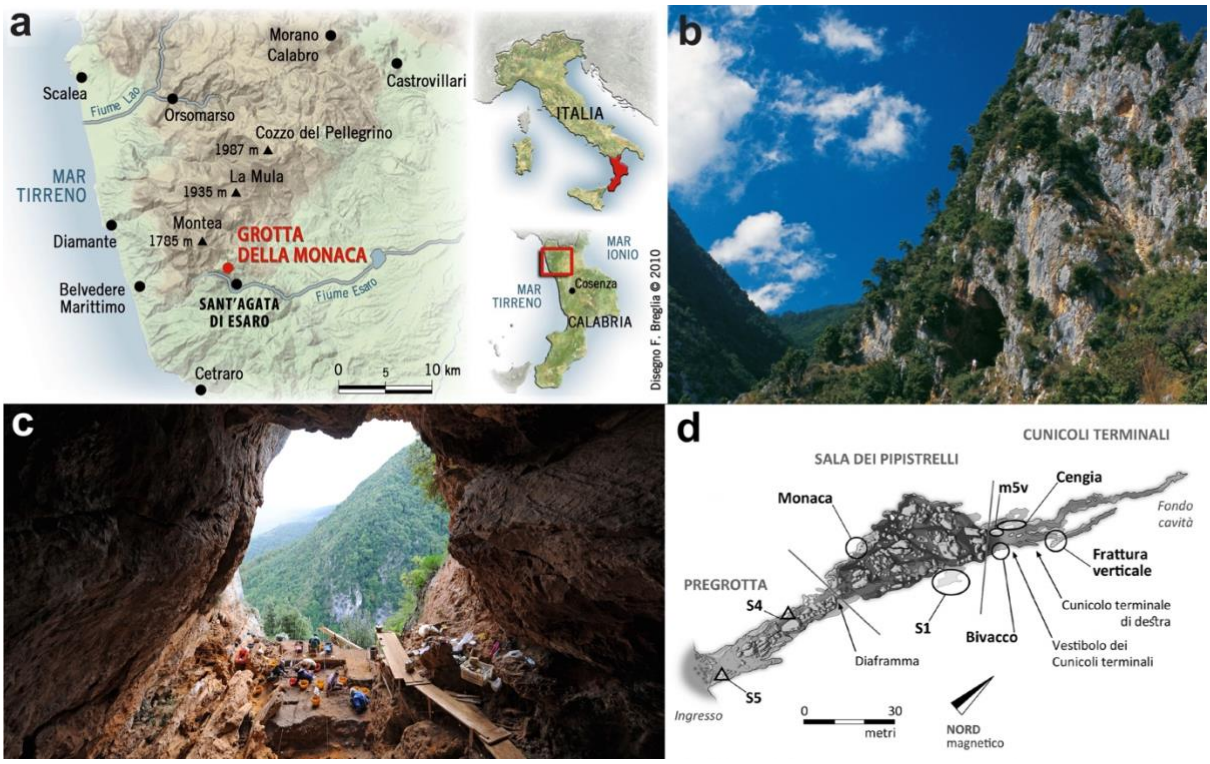

Supplementary Figure 1. Geographic and archaeological context of Grotta della Monaca. **a)** Location of Grotta della Monaca within the territory of the Calabria region (drawing by Francesco Breglia); **b)** The entrance to Grotta della Monaca at the top of a limestone massif (photo by Felice Larocca); **c)** The wide entrance of the cave seen from the inside during an archaeological excavation campaign (photo by Felice Larocca); **d)** Plan of Grotta della Monaca showing the layout and the location of the different archaeological subsectors mentioned in the text. Adapted from an original graphic elaboration by Francesco Breglia and Felice Larocca.

From this point, the "Cunicoli Terminali" begins, constituting the furthest subterranean reaches of the cave that splits in numerous branches delving almost 110 meters from the entrance of the cavity. The archaeological investigations conducted in the innermost area of the "Sala dei Pipistrelli" and the "Cunicoli Terminali" have unearthed stone walls, stone tools and working footprints on the rock walls that led to identify the area as subjected to mining exploitation. The deposits of ferrous hydroxides were clearly exploited by humans already during the Palaeolithic, as evidenced by the discovery of flint and bone tools in proximity to the goethite veins closest to the "Pregrotta" area[1]. Radiocarbon dates place the point of maximum expansion of copper mining activities between the end of the V and the beginning of the IV millennium BCE. The ultimate abandonment of prehistoric mining activities seems to have happened in the second half of the IV millennium BCE[2].

Supplementary Table 1. Complete list of radiocarbon dates available from Grotta della Monaca.

**ID analysis**	**Individual**	**CODE (Year)**	**Site (area)**	**Institute of dating**	**Radiocarbon Age (BP)**	**Calibrated Age (2σ)**
Unknown	Unknown	Unknown (2004)	Grotta della Monaca (Ct.dx/fv)	University of Groningen	Unknown	1690 BCE (95.4%) 1440 BCE
Unknown	Unknown	Unknown (2004)	Grotta della Monaca (Ct.dx/fv)	University of Groningen	Unknown	1779 BCE (95.4%) 1520 BCE
MONACA11	Individual 5 (GDM5)	LTL5084A (2008)	Grotta della Monaca (m5v)	CEDAD Università del Salento	3204±45	1610 BCE (95.4%) 1400 BCE
MONACA12	Individual 6 (GDM6)	LTL5085A (2008)	Grotta della Monaca (m5v)	CEDAD Università del Salento	3263±45	1640 BCE (95.4%) 1430 BCE
MONACA14	Individual 12 (GDM12 / GMO019)	LTL5087A (2008)	Grotta della Monaca (m5v)	CEDAD Università del Salento	3192±45	1560 BCE (92.1%) 1380 BCE
MONACA15	Individual 14 (GDM14)	LTL5088A (2008)	Grotta della Monaca (m5v)	CEDAD Università del Salento	3247±45	1630 BCE (95.4%) 1420 BCE
MONACA16	Individual 17	LTL5089A (2008)	Grotta della Monaca (m5v)	CEDAD Università del Salento	3308±50	1700 BCE (91.7%) 1490 BCE
MONACA17	Individual 11 (GDM11 / GMO018)	LTL5090A (2008)	Grotta della Monaca (m5v)	CEDAD Università del Salento	3270±45	1670 BCE (95.4%) 1430 BCE
GROTTA DELLA MONACA 1	Unknown	LTL22118 (2022)	Grotta della Monaca (b)	CEDAD Università del Salento	4962±45	3804 BCE (86.3%) 3643 BCE

GROTTA DELLA MONACA 2	Unknown	LTL22119 (2022)	Grotta della Monaca (b)	CEDAD Università del Salento	3215±45	1546 BCE (95.4%) 1406 BCE
GDM104	Unknown individual (GDM104 / GMO005)	LTL33400 (2024)	Grotta della Monaca (m5v)	CEDAD Università del Salento	3259±40	1616 BCE (95.4%) 1442 BCE
GDM106	Unknown individual (GDM106 / GMO007)	LTL33401 (2024)	Grotta della Monaca (m5v)	CEDAD Università del Salento	3336±40	1692 BCE (95.4%) 1512 BCE

Several excavation campaigns conducted since 2000 by the Regional Center of Speleology "Enzo dei Medici" and the University of Bari have documented the existence of an extensive hypogeum necropolis in the most underground areas of the cavity. The utilization of the cave as a funerary area dates back to the mid-second millennium BCE, a period when mining activities within the cave had likely ceased. Unfortunately, no settlements associated with the cemetery were ever unearthed by the archaeologists nearby the site. Plausibly, one or more Bronze Age villages existed in the valley a few kms from the entrance of the cavity, where the modern village of Sant'Agata d'Esaro lies nowadays. Scattered skeletal remains were discovered in the innermost chambers of the cavity, in niches and fractures of the walls that likely host multiple burials. Most of the osteological material was found by the archaeologists as heaps of bones grouped in disordered piles in sector *m5v*, that follow the natural sloping course of the cave floor[3] (Supplementary Fig. 2a-2b). The skeletal material recovered in sector m5v was not associated with any funerary assemblage. Only a few artifacts were found in the immediately adjacent area, and these appear to represent votive offerings rather than grave goods. They include a bowl, a jar, and a spindle whorl. All these artifacts are exclusively attributable to the Protoapennine facies.

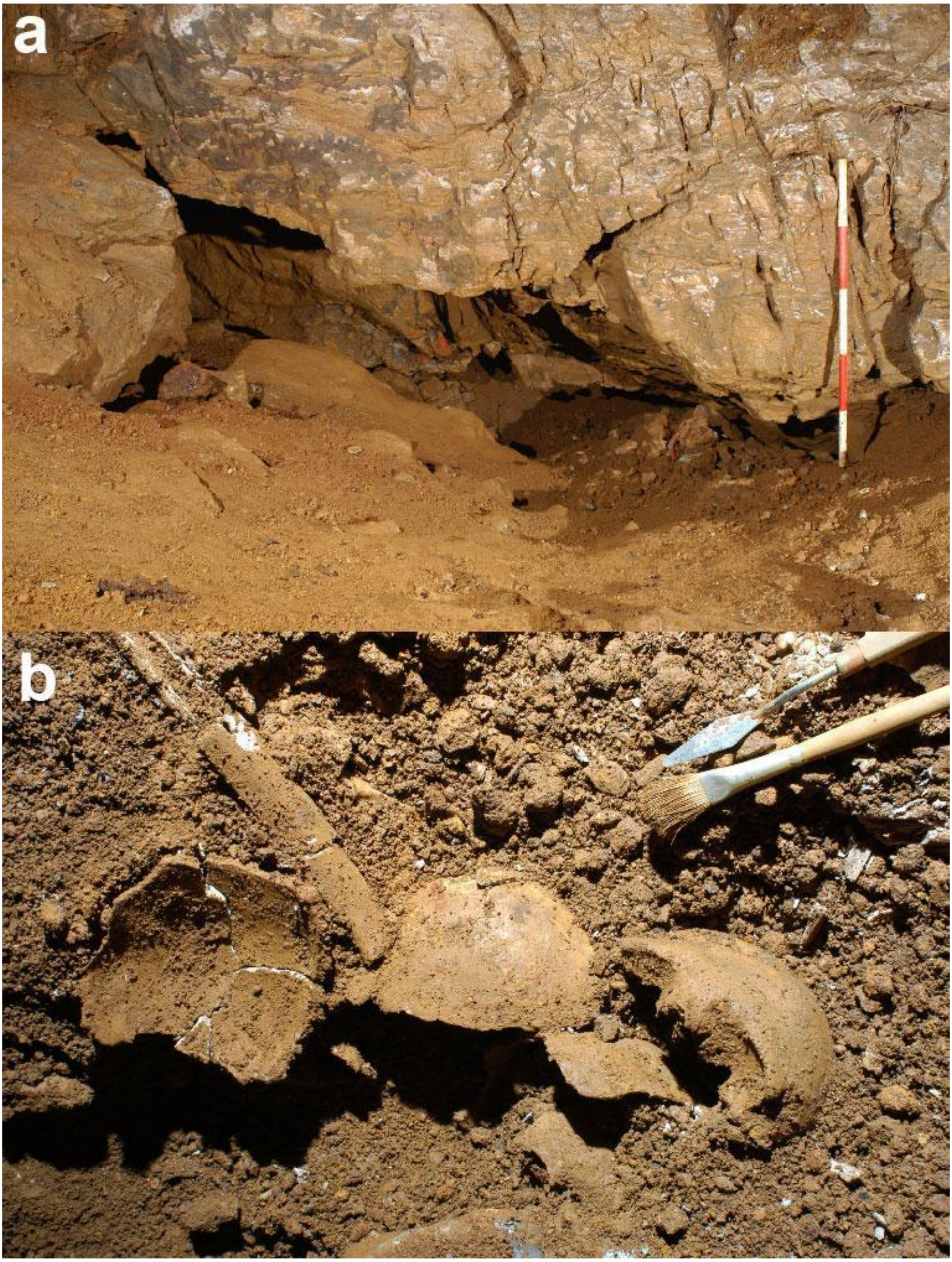

Supplementary Figure 2. The “m5v” sector before and during excavations. **a)** The entrance to the archaeological sector “m5v”sector, as it appeared before the excavation activities; **b)** A phase of the recovery of human bone remains within the “m5v”sector (photographs taken by Felice Larocca).

The chronological sequence of the material recovered outside of *m5v* is difficult to interpret: both the skeletal remains and ceramics are in a poor state of preservation due to multiple taphonomic factors, including the cave's high humidity, persistent disturbance by fauna, and extensive post-medieval anthropic activity. In the absence of a well-defined stratigraphy, it is nonetheless possible to identify clusters of ceramic material, which likely represent the outcome of post-depositional processes such as mining activity, trampling, and the displacement of artifacts into fractures or niches in the bedrock. Preliminary chrono-typological observations suggest the presence of three phases rather than two: the above mentioned Protoapennine, the Piano Conte-type materials, and the Laterza-type elements. A comprehensive re-evaluation of the ceramic assemblage from the cave is currently being undertaken as part of a doctoral project at the University of Bari. Beyond *m5v*, at least 12 areas of the site have yielded sparse and commingled human remains. Due to the chaotic dispersal of the material and the absence of a clear stratigraphy, it is not possible to define these areas as formal funerary contexts. These areas are defined as:

- PMs/S4: *Pregrotta Media superiore* (Excavation trench nr. 4).
- SP/S1: *Sala dei Pipistrelli* ("Hall of Bats", Excavation trench nr. 1). The burial area SP1 develops along the right side wall of the "Sala" and appears as a deepening created along the detachment of a mighty limestone boulder.
- CTv/b: *Vestibolo dei cunicoli terminali*. The so-called *Bivacco* (*b*) corresponds to a flat area in front of the terminal tunnels, near the right wall of the cavity. Human remains recovered from this sector derive from the peripheral zones along the wall and consist of disarticulated anatomical elements in a highly of fragmented state. Ceramic artifacts from this area include material attributable to both the Eneolithic and the Bronze Age, reflecting the 'incoherent' nature of the context. A similar pattern is evident in the sporadic and scattered skeletal remains, which also date to both the Eneolithic and the Bronze Age (Supplementary Table 1).
- CTv/S7: *Vestibolo dei cunicoli terminali* (Excavation trench nr. 7). This is an area which, from the left wall, slopes down towards a flatter area directly in front of the terminal tunnels, reaching the right wall of the cave. The sampled bone remains mostly refer to phalanges and teeth and to anatomical parts that are not connected and very fragmented.

- CTsx.i: *Cunicolo terminali di sinistra*. It is a narrow conduit, approximately 20 meters in length, descending steeply with a negative elevation difference of about 12 meters from the entrance to the cave, and characterized by a substantial accumulation of clasts on the floor. Sporadic bone remains were recovered near the entrance of the conduit, in a lateral recess along the left wall. In the same area, highly fragmented ceramics were found, which have attributed to Eneolithic (Laterza) and Middle Bronze Age (Protoapennine) horizons.
- CTdx/fv: *Cunicolo terminale di destra* - *Frattura verticale* (vertical fracture) is a narrow, deep fissure located approximately 15 meters from the entrance of the terminal tunnel on the right side. The archaeological material was recovered from pockets and depression at levels along the fracture, having accumulated on an earthen slope that descends to the base. The scarce assemblage includes fragments of vessels, among which a ceramic olla can be attributed to the Bronze Age, and several human skeletal remains, including highly fragmented teeth, ribs, femora and vertebrae.
- CTv/S8: Vestibolo dei cunicoli terminali (Excavation trench nr. 8). This area is located on a sub-horizontal rocky step that overlooks the rocky peak at the base of which originates the entrance to the crack that leads into m5v.

Supplementary Note 2: Anthropological remains

(Francesco Fontani, Felice Larocca and Fabiola Arena)

Anthropological analyses of the human remains sampled for this study were carried out at the Laboratory of Archaeo-Anthropology and Forensic Anthropology of the University of Ferrara (Italy). When possible, age assessment was done on the basis of morphology of the auricular surface of the os coxae[4,5], pubic symphysis surface changes[6], synchondrosis of ectocranial suture[5,7] and tooth wear[4,8]. Following standard criteria[7], we divided the specimens into three age categories: young adults (20–35 years), middle adults (35–50 years), old adults (50+). Sex diagnosis was based on skull and pelvis morphology[9] and osteometric characters of the skull[10,11], mandible[12], scapula[13], humerus[14–17], ulna[18], femur[16,19]), tibia[14,15] and talus[20]. We established a minimum number of 24 individuals, with a greater frequency of infants aged between

0 and 12 years (62.5%), among which three neonatal children. Due to the extreme commingled nature of the remains, only for 10 individuals it was possible to determine sex, and in only six cases the anthropologists were able to reconstruct the almost complete skeletons of the individuals. Among the many sectors where sporadic evidence of human remains was found, burial ground *m5v* represents the only proper funerary area recognised inside the cavity. Here, piles of commingled bones were found, some of which were still in anatomical connection[3] and identified as secondary depositions. Five individuals were recognized on the basis of the skull (one elderly individual and four young adults), ten individuals based on the left humerus (infants class 1, 2, 3), four on the basis of the right humerus (three adolescents and one young individual of unspecified age), three individuals based on the right pubis (infants of class 1), and two newborn (or dead at birth) individuals based on the tibia. Palaeopathological analyses highlighted the widespread presence of *cribra* (*cranii* and *orbitalia*), probably caused by the persistence of anaemic states related to iron and vitamin deficiency, but also to poor living conditions, associated with malnutrition, scarce hygiene and persistence of diseases. The anthropological analysis carried out on dental diseases showed signs of a diet characterized by a low intake of meat and a high consumption of leathery foods such as seeds and grains. Interestingly, three female subjects presented an anatomical alteration of the tooth of the epistrophe that is bifid or not welded. This trait, due to a defect of ossification, represents a rare alteration that may reflect some form of relatedness between individuals. Such evidence led to speculating regarding the possibility of kinship-related forms of social organization of the funerary ground, possibly related to a specific Middle Bronze community.

We selected the following samples for ancient DNA analysis and provide archaeo-anthropological information for each, including loose skeletal elements that could not be determined as belonging to a specific individual.

GMO001. Archaeological ID: GDM10. Isolated left petrous bone associated with known individual 10, found in sector *m5v* and associated to a +30 years at death female individual. The individual presented a severe case of neoplasm: a deep sulcus was observable on the endocranial side of the left parietal bone, as a result of a meningioma. The individual lacks growth of the right and left second lower premolars,

whose sockets were still occupied at the moment of death by the corresponding deciduous teeth (right and left second molars).

GMO002. Archaeological ID: GDM101. Isolated left petrous bone found in sector *b*. Based on anthropological analysis, the sample was indicated to come from an adult subject, but it was not possible to associate this sample with any of the known individuals.

GMO003. Archaeological ID: GDM102. Isolated left petrous bone found in sector *fv*. Based on anthropological analysis, the sample was indicated to come from an adult subject, but it was not possible to associate it with any of the known individuals.

GMO004. Archaeological ID: GDM103. Isolated left petrous bone found in sector *m5v*. Based on anthropological analysis, the sample was indicated to come from an infant or pre-adolescent subject, but it was not possible to associate it with any of the known individuals.

GMO005. Archaeological ID: GDM104. Isolated left petrous bone found in sector *m5v*. Based on anthropological analysis, the sample was indicated to come from an adult subject, but it was not possible to associate it with any of the known individuals.

GMO006. Archaeological ID: GDM105. Isolated left petrous bone found in sector *m5v*. Based on anthropological analysis, the sample was indicated to come from a infant subject, but it was not possible to associate it with any of the known individuals.

GMO007. Archaeological ID: GDM106. Isolated left petrous bone found in sector *m5v*. The age estimation of this isolated petrous bone was tentatively assessed on comparison with the petrous bones of individuals of known age from the same context. The sample was indicated to come from an adolescent or juvenile subject, but it was not possible to associate it with any of the known individuals.

GMO008. Archaeological ID: GDM107. Isolated left petrous bone found in sector *m5v*. The age estimation of this isolated petrous bone was tentatively assessed on comparison with the petrous bones of individuals of known age from the same context.

The sample was indicated to come from an adolescent or juvenile subject, but it was not possible to associate it with any of the known individuals.

GMO009. Archaeological ID: GDM108. Second upper left permanent molar found in sector “CTsx.i”. The tooth showed the presence of caries, but it was not possible to estimate age, sex or associate it with any of the known individuals.

GMO010. Archaeological ID: GDM109. Second upper left permanent premolar found in sector *S7*. It was not possible to estimate age, sex or associate it with any of the known individuals.

GMO011. Archaeological ID: GDM110. First upper left permanent incisive found in sector *S7*. It was not possible to estimate age, sex or associate it with any of the known individuals.

GMO012. Archaeological ID: GDM111. First lower right permanent molar found in sector *S7*. It was not possible to estimate age, sex or associate it with any of the known individuals.

GMO013. Archaeological ID: GMO013, First upper left deciduous molar found in sector *fv*. It was not possible to estimate age, sex or associate it with any of the known individuals.

GMO014. Archaeological ID: GDM113. First upper left permanent premolar found in sector *b*. It was not possible to estimate age, sex or associate it with any of the known individuals.

GMO015. Archaeological ID: GDM115. Lower right deciduous canine found in sector *S7*. It was not possible to estimate age, sex or associate it with any of the known individuals.

GMO016. Archaeological ID: GDM116. Second lower right premolar found in sector *S8*. It was not possible to estimate age, sex or associate it with any of the known individuals.

GMO017. Archaeological ID: GDM117. Second lower left premolar found in sector *S4*. It was not possible to estimate age, sex or associate it with any of the known individuals.

GMO018. Archaeological ID: GDM11B. Isolated left petrous bone from known individual 11. The remains were found in 2008 in sector *m5v*. The individual was indicated as a young female adult of +30 years at death. A severe case of periostitis was observed on the intercranial side of the left parietal, to such an extent that the trabecular bone was corroded by pus expulsions as the result of non-specific infections not associable to microorganisms. A particular feature of individual 11, possibly related to this infection state, is the presence of a trepanation, practiced by human hand on the left parietal bone. The presence of bone callus on all margins of the fracture shows that the woman survived for at least two months after surgery. However, adjacent to the hole, on the intracranial side of the left parietal, inflammation of the periosteum, degenerated into an infection, is evident. It may have formed later in the area adjacent to the perforation and caused the death of the individual after the formation of the bone callus; or might have been the reason for the operation. Direct radiocarbon dating was performed on a fragment of the skull, and the individual dated back to 3270 ± 45 BP. The corresponding calibrated dating is equal to 1670 (95.4%) 1430 BCE (2 confidence level, OxCal v3.10). Mitochondrial data were previously investigated[21].

GMO019. Archaeological ID: GDM12B. Isolated right petrous bone found in sector *m5v* and associated with known individual 12. The individual was a female young adult (~20 years old at death) on the basis of cranial features. A humerus sample from the individual was directly radiocarbon dated to 1560 BCE (92.1%) 1380 BCE (calibrated dates, 2 confidence level, OxCal v3.10). Mitochondrial data were previously investigated[21].

GMO020. Archaeological ID: GDM22. Isolated right petrous bone found in sector *m5v* and associated with known individual 22. Due to the scarcity of osteological material, age at death was estimated on the basis of left humerus and reported an age between 8 and 15 years at death.

GMO021. Archaeological ID: GDM23. Isolated right petrous bone found in sector *m5v*. On the basis of cranial features, the individual was estimated to be an infant of 1-3 years at death, but it was not possible to associate it with any of the known individuals.

GMO022. Archaeological ID: GDM24. Isolated left petrous bone found in sector *m5v* and associated with known individual 24. The individual, estimated to be a male of +30 years at death, reported to be affected by neoplasm on the basis of rounded lesions on the occipital bone probably resulting from metastases activity. Mitochondrial data were previously investigated[21].

GMO023. Archaeological ID: GDM7B. First upper left deciduous molar associated with known individual 7. The remains of the individual were found in 2008 on sector *m5v*, and anthropological analysis reported the individual to be an infant (class 2) of <7 years at death, indicated as male on the basis of pelvis and cranial features. The individual was indicated as affected by "cribrotic syndrome", in this case due to the coexistence of *cribra orbitalia*, *cribra humeri* and *cribra femora*. On the intercranial side of the frontal bone, evidence of non-specific infection was observed by the anthropologists and associated with the presence of bone metastasis possibly associated with extra-osseous neoplasm. Mitochondrial data were previously investigated[21].

Supplementary Note 3: Uniparentally inherited haplogroups

(Francesco Fontani and Alissa Mittnik)

Mitochondrial DNA haplogroups After reconstructing the consensus mitochondrial genomes from each individual as described in the Method section of the Main Text, we used Haplogrep3 and visual inspection on IGV to reconstruct individuals' mitochondrial phylogeny. GMO001 was assigned to the mitochondrial haplogroup K1a3, with the assignment being sustained by 20 defining variants in relation to the rCRS. GMO018 was associated with haplogroup K1a2a on the basis of 27 defining variants, revealing a lineage that appeared to be widespread in Central and Southern Europe during the Neolithic and Chalcolithic periods. The diversification within the dataset was marked by the presence of seven individuals that were associated with

branches of the clade H, a clade that had likely originated around 30000-25000 years ago in the Near East. Individual GMO006 (12 variants) is haplogroup H5a+152C, a subgroup of clade H5 solely reported in a Chalcolithic individual from Croatia[22]. Individual GMO015 (11 variants) and GMO022 (6 variants found through visual inspection on IGV) share haplogroup H1e, a lineage fairly common in Southern Europe during the Bronze Age, witnessed also in Middle Neolithic to Chalcolithic individuals from Sicily[23,24]. For GMO012, 8 variants were found to associate the individual to haplogroup H2c. GMO009, distinguished by 6 variants, was assigned to haplogroup H3am, also present in a Bronze Age sample from Central Italy[25]. Despite being constrained by very low coverage, individual GMO004 was tentatively associated with the major haplogroup H, on the basis of two defining variants. Based on 28 informative variants, GMO007 was assigned to haplogroup J1c1, a widespread lineage in Europe since the Neolithic, while GMO013 was assigned to haplogroup T2c1 (15 variants), which appears to have a Near Eastern origin around the Last Glacial Maximum. Notably, the presence of T2 lineages were found in high percentage among the Yamnaya people[26–28], while the most ancient samples reporting T2c1 haplogroup were reported from Neolithic Anatolia[29]. Two individuals reported mitochondrial lineages related to the major haplogroup U, an early-diverging branch of the human mitochondrial phylogeny that arose approximately 60000 years ago. GMO010 was assigned to haplogroup U8b1b1 (based on 28 variants), which finds a fairly high distribution in Neolithic and Early Bronze Age Sicilians[24,30], but also in Neolithic Central Europe populations. Finally, GMO011 exhibits 25 variants associated with haplogroup U1a1a, which was recently reported also in a Sardinian Neolithic sample[24].

Y chromosome haplogroups Y-chromosome haplogroups were assigned by identifying specific variants listed in the ISOGG Y-DNA tree (https://isogg.org/tree/). Haplogroup determination followed a stepwise, root-to-tip evaluation of diagnostic SNPs based on approaches described in the main text. To increase confidence in haplogroup calls, the analysis prioritized transversion-derived reads, initially excluding transitions. After the preliminary assignment, high-coverage transition-derived reads were reviewed to confirm or refine the results.

Sample GMOO04 exhibited low Y-chromosome coverage. The most reliable assignment is haplogroup G2~, supported by three transversion reads indicating a G lineage. Additionally, one transversion read each supported the downstream branches

G2a2b2a (PF3330) and G2a2b2a1a1b1a1~ (Z753), suggesting a tentative placement within this clade.

A total of 10 transversion reads supported a broad I2~ lineage for GMO006, with 7 of these also consistent with I2a. The most likely assignment is I2a1b, backed by eight transversion SNPs. Further refinement points toward I2a1b2, with one transversion read on the defining SNP SK1262/Y11315, supported by three additional transition reads on FGC29562/Y10705 and S2599.

Individual GMO007 is assigned to haplogroup H2a1~, primarily supported by 12 transversion reads indicating an H2~ background. The defining SNP Z18988 is covered by three transversion reads, while SNP Z18874 has one. Six additional transitions also support the H2a1~ placement.

For individual GMO009, the most confident classification is R1b1b2a. This is supported by five transversion reads targeting key SNPs: FGC20992/V1732 (3 reads), FGC20991/Y8446 (1), and FGC21010/Y8449 (1). The assignment is further corroborated by 15 transition-derived reads.

Individual GMO015 sample is confidently assigned to haplogroup R1b1a1b, based on nine transversion reads covering SNPs CTS8591/PF6477 (3 reads), L1348/PF6521/YSC0000238 (1), PF6430 (1), PF6433 (2), and PF6497/YSC0000219 (1). An additional 18 transition reads reinforce this classification.

Finally, individual GMO022 has low Y-chromosome coverage. The most conservative assignment being haplogroup F, based on two transversion reads at SNP L470/M3735/PF2730. However, due to the parent-offspring relationship with individual GMO007, H and H2~ clades were also explored. Two transition reads on SNP SK1189 support a tentative assignment to H2~.

Supplementary Note 4: PCA for capture and PMD strategy assessment

(Francesco Fontani)

To assess potential discrepancies in genetic variation introduced by different enrichment protocols, we conducted a principal component analysis on ancient DNA libraries that were processed independently using both 1240K and Twist enrichment methods. Specifically, we analysed samples GMO001, GMO009, GMO010, and GMO012 to explore the possibility of variation patterns due to induced enrichment

strategy biases. To ensure robustness and minimize protocol-induced bias, we filtered the dataset to retain only a subset of SNPs identified as less susceptible to allelic bias. We followed an approach which targeted a SNP set of 469,332 SNPs that significantly reduced the observed biases in population genetic analyses across different library types[31]. Our results (Supplementary Fig. 3) support the co-analysability of data enriched via the Twist assay with 1240K enriched data for GMO001, GMO010 and GMO012, which all positioned homogeneously in the PCA, with respect to the library strategy employed. Sample GMO009 is the only one for which discrepancies are visible between 1240K and Twist enriched data, with the former showing a visible attraction towards European Neolithic populations compared to the Twist enriched form. Given evidence of potential contamination in the sample (see Methods), we retained only post-mortem damaged reads and projected the sample on the PCA, showing that even at lower resolution, the individual is genetically affine to the cluster when only deaminated sequences are retained.

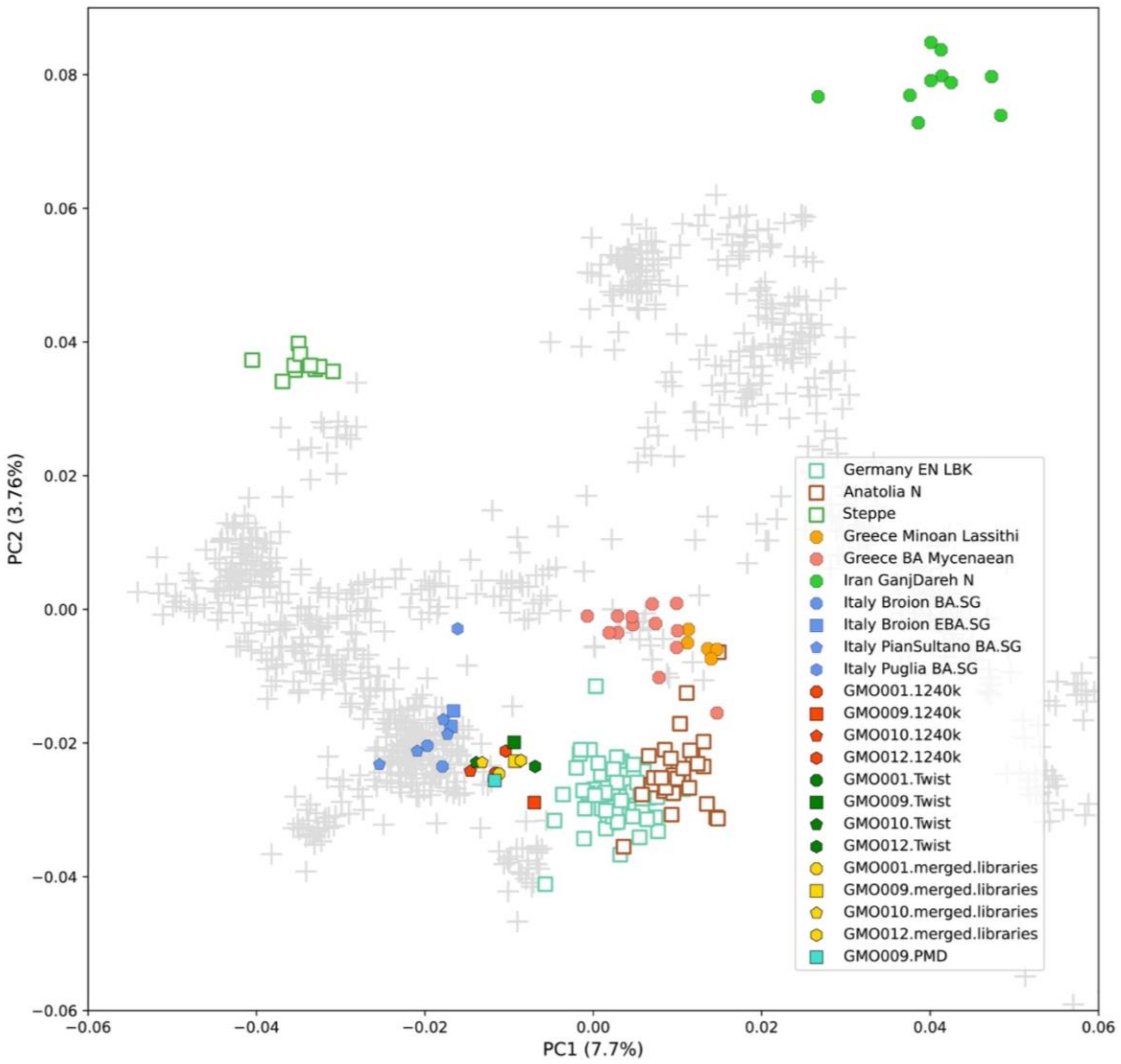

Supplementary Figure 3. Comparative PCA for Twist, 1240K captured and PMD-filtered data using a restricted set of co-analysable SNPs. The Principal component analysis is calculated on the same set of present-day western Eurasians (n=1074) from the Human Origin Affymetrix dataset used for the PCA in the main text; the projected ancient individuals (n=158) are displayed as coloured shapes.

Consistently high f3 values, obtained after additional f3-statistics in the form $f3$(Mbuti.DG; X, Y), demonstrate that the different libraries carry near-identical genetic information (Supplementary Table 2). We additionally computed f4-statistics of the form $f4$(Sample1_Twist, Sample1_1240k; Sample2_Twist, Sample2_1240k) for all sample pairs with data from both capture protocols. All resulting Z-scores (Supplementary Table 3) ranges between -0.91 and 0.88, indicating no systematic attraction or bias related to enrichment method.

Supplementary Table 2. Results from f3-statistics in the form f3(Mbuti; X, Y). Here, X and Y represent either 1240k, Twist-enriched or merged libraries from the same individual. Strongly positive f_3 values and high Z-scores confirm consistent ancestry signals across capture methods and library types, indicating no evidence of bias.

**Pop1**	**Pop2**	**Pop3**	**f3**	**Z**
Mbuti.DG	GMO001.1240k	GMO001.merged.libraries	8,35597E+13	141,818
Mbuti.DG	GMO009.1240k	GMO009.merged.libraries	3,58927E+13	101,17
Mbuti.DG	GMO010.1240k	GMO010.merged.libraries	4,24402E+13	102,987
Mbuti.DG	GMO012.1240k	GMO012.merged.libraries	9,59683E+13	160,683
Mbuti.DG	GMO001.1240k	GMO001.Twist	9,90705E+13	152,405
Mbuti.DG	GMO009.1240k	GMO009.Twist	4,65553E+13	117,373
Mbuti.DG	GMO010.1240k	GMO010.Twist	5,51032E+13	125,729
Mbuti.DG	GMO012.1240k	GMO012.Twist	1,13549E+14	165,745
Mbuti.DG	GMO001.Twist	GMO001.merged.libraries	2,64684E+14	223,949
Mbuti.DG	GMO009.Twist	GMO009.merged.libraries	2,03984E+14	202,644
Mbuti.DG	GMO010.Twist	GMO010.merged.libraries	2,04144E+14	203,382
Mbuti.DG	GMO012.Twist	GMO012.merged.libraries	2,58996E+14	211,24

Supplementary Table 3. Results from f4-statistics in the form f4(Sample1_Twist, Sample1_1240k; Sample2_Twist, Sample2_1240k). Statistics were computed for all possible pairs of individuals with both Twist and 1240k data. Z-scores falling within the range of -2 to +2 indicate the absence of systematic bias introduced by differing capture methods.

**Pop1**	**Pop2**	**Pop3**	**Pop4**	**f4**	**Z**
GMO001.Twist	GMO001.1240k	GMO009.Twist	GMO009.1240k	-0,0017	-0,91
GMO001.Twist	GMO001.1240k	GMO010.Twist	GMO010.1240k	-0,0000	-0,00
GMO001.Twist	GMO001.1240k	GMO012.Twist	GMO012.1240k	-0,0004	-0,411
GMO009.Twist	GMO009.1240k	GMO010.Twist	GMO010.1240k	0,0012	0,463
GMO009.Twist	GMO009.1240k	GMO012.Twist	GMO012.1240k	0,0014	0,88
GMO010.Twist	GMO010.1240k	GMO009.Twist	GMO009.1240k	0,0012	0,463
GMO010.Twist	GMO010.1240k	GMO012.Twist	GMO012.1240k	-0,0004	0,282

Supplementary Note 5: Clustering individuals via pairwise qpWave

(Francesco Fontani)

We used qpWave from ADMIXTOOLS to cluster individuals from similar archaeological contexts. We used a p>0.01 threshold as a criterion for clustering, *allsnps:YES*, and the following "Right" populations from the same dataset we used in qpAdm (Supplementary Data 17): WHGA, WHGB, Anatolia N, Balkan N, Steppe, Afanasievo, Israel Natufian. The consistency of the method was tested by performing pairwise qpWave on a subset of 55 individuals, composed of 11 individuals from Grotta della Monaca and 44 relevant ancient individuals from Neolithic to Bronze Age Italy. If the resulting p-value, calculated by the qpWave program using a likelihood ratio approach, was lower than 0.01, it was interpreted as a poor fit of the proposed model for the tested pair. Among the GMOs, most of the individuals passed the qpWave analysis at the p-value > 0.01 threshold and they are consistent in forming a clustered genetic group, namely "GMO". All the pairs tested involving GMO010 and any other GMO individual showed poor fits of the model. Hence, we labelled this individual as a genetic outlier and kept it separated from the main cluster.

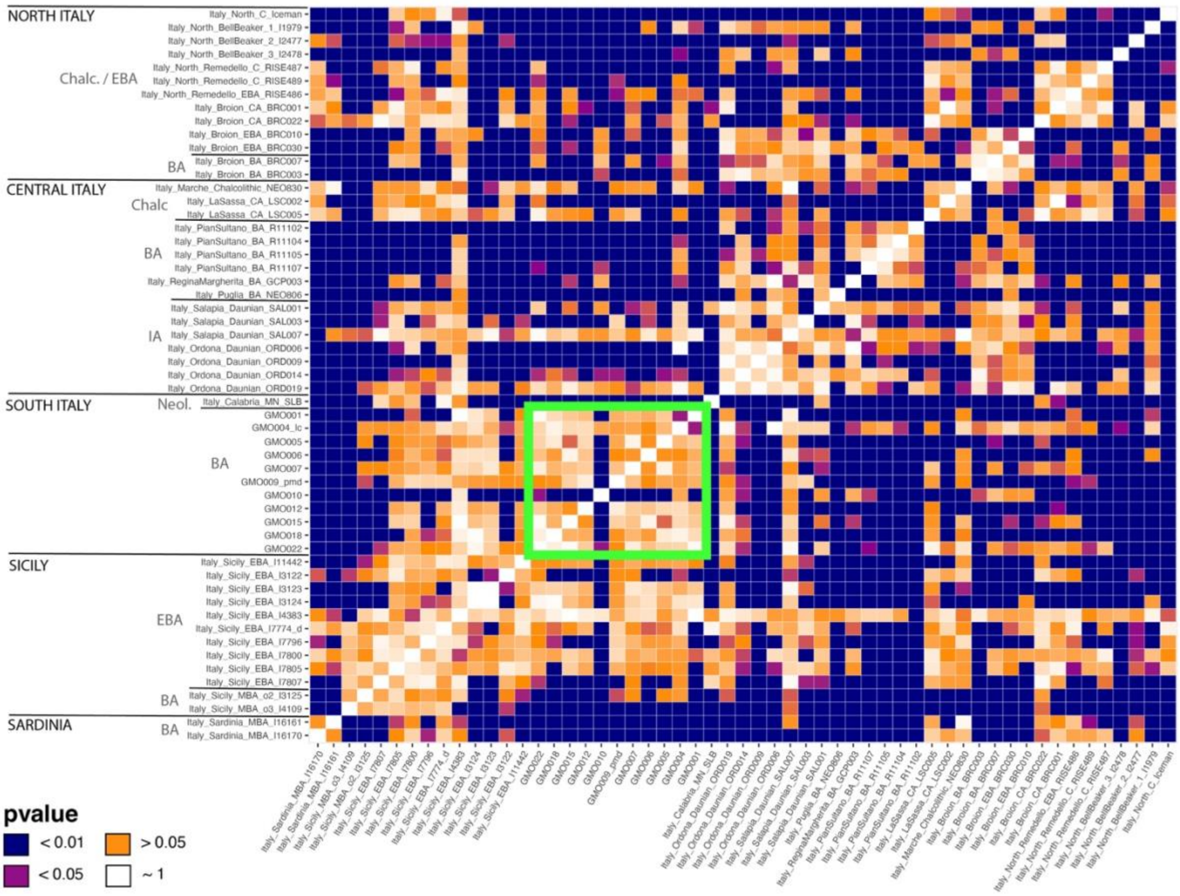

Supplementary Figure 4. Heatmap of pairwise qpWave clustering results. Individuals are broadly grouped by geographic provenance and ordered chronologically within each region. Blue indicates pairs for which the qpWave model was rejected at $p < 0.01$.

Supplementary Note 6: qpAdm modelling

(Francesco Fontani)

To reconstruct the Grotta della Monaca gene pool and its ancestral relation with coeval European populations, we first applied a parsimonious approach, as extensively described in recent publications[32–34]. We calculated the proportion of ancestry for distal using minimal source populations, and when we could not find a plausible model, we added relevant populations. We also applied a rotating approach, to test our samples for relevant population models, in 2 ways, 3 ways, 4 ways and 5 ways models. For 2 way models, we used the following 4 populations as our base "Right" set: OldAfrica (n=9), WHGB (n=41), Anatolia N (n=24), Afanasievo (n=24). Our base 2 way "Left" ancestral population set was composed by hunter-gatherers individuals from

Mesolithic Western Europe (WHGA, n=13) and Neolithic farmers from the Balkan peninsula with minimal hunter–gatherer admixture (Balkan N, n=21). For 3 way models, we added individuals associated with the Afanasievo population (n=24) to the "Right" set, and Yamnaya pastoralists (Steppe, n=18) to the "Left" set as a representative of the steppe-herders ancestry. Additionally, we tested alternative 3 way models for pre-Bronze Age population requiring a more Levantine ancestry by adding either hunter-gatherers individuals from the Caucasus (CHG, n=2) or Neolithic farmers from Iran (Iran N, n=8) to the "Right" and "Left" set, employing the rotating approach described above. Presence of such ancestry was also tested on a 4 way model requiring an additional Levantine source along with the base 3 way model. An alternative 4 way model was employed to test for the presence of North African Ancestry, adding the Iberomaurusian individuals (Morocco Iberomaurusian.AG, n=6) to the "Right" set and a subset of Neolithic farmers from North Africa (OldNorthAfrica, n=10) to the "Left" set.

For proximal modelling, we used as a base "Right" set the same "Right" populations that were used for the distal modeling, but also added in the five sources of the distal modeling, so that the final proximal "Right" set was composed of: OldAfrica, WHGB, Anatolia N, Afanasievo, WHGA, Balkan N, Steppe, CHG, Iran N. Leveraging the limited data available for Neolithic individuals from the Italian peninsula and surrounding islands, we used Italy Lazio N.SG (n=4), Italy Marche N.SG (n=1), Italy Sardinia N (n=15), Italy Sicily MN (n=6), Italy Sicily Stentinello (n=5), Italy Calabria MN (n=1) and Italy South N.SG (n=5) as a plausible source to assess local farmer ancestry. Additionally, we incorporated Early Bronze Age individuals from either northern Italy or Sicily to trace for a local origin of Steppe ancestry components, using Italy Broion EBA.SG (n=2) and Italy Sicily EBA (n=7) as sources. The GMO main genetic cluster was also tested as a source of local ancestry to model the genetic composition of the outliers. For both the distal and proximal approaches, we kept the best working model which required the fewest sources of population.

Supplementary References

1. Breglia, F., Caricola, I. & Larocca, F. Macrolithic tools for mining and primary processing of metal ores from the site of Grotta della Monaca (Calabria, Italy). *JLS* **3**, 57–76 (2016).
2. Larocca, F. & Levato, C. From the imprint to the tool: the identification of prehistoric mining implements through the study of digging traces. The case of Grotta della Monaca in Calabria (Italy). in *The Impact of Historic and Prehistoric Mining Activities on the Environment and Socities in Europe* (Innsbruck, 2012).
3. Arena, F. & Gualdi-Russo, E. Il sepolcreto protostorico di Grotta della Monaca in Calabria. Aspetti antropologici. in *XX Congresso AAI Ferrara. Annali online dell'Università degli Studi di Ferrara* 74–80 (2014).
4. Lovejoy, C. O., Meindl, R. S., Pryzbeck, T. R. & Mensforth, R. P. Chronological metamorphosis of the auricular surface of the ilium: A new method for the determination of adult skeletal age at death. *Am. J. Phys. Anthropol.* **68**, 15–28 (1985).
5. Meindl, R. S. & Lovejoy, C. O. Ectocranial suture closure: A revised method for the determination of skeletal age at death based on the lateral-anterior sutures. *American J Phys Anthropol* **68**, 57–66 (1985).
6. Todd, T. W. Age changes in the pubic bone. I. The male white pubis. *American J Phys Anthropol* **3**, 285–334 (1920).
7. Buikstra, J. E. & Ubelaker, D. H. *Standards for Data Collection from Human Skeletal Remains*. (Arkansas archeological survey, Fayetteville (Ark.), 1994).
8. Brothwell, D. R. *Digging up Bones: The Excavation, Treatment, and Study of Human Skeletal Remains*. (Cornell University Press ; British Museum (Natural History), Ithaca, N.Y. : [London], 1981).
9. Acsadi, G. T. & Nemeskéri, J. *History of Human Life Span and Mortality*. (Budapest : Akademiai Kiado, 1970).
10. Giles, E. & Elliot, O. Sex determination by discriminant function analysis of crania. *American J Phys Anthropol* **21**, 53–68 (1963).
11. Demoulin, F. Importance de certaines measures craniennes (en particulier de la longueur sagittale de la mastoide) dans la determination sexuelle des cranes. *Bull Mem Soc Anthropol Paris* **9**, 259–264 (1972).

12. Piquet, M. M. Étude sur la robustesse de la mandibule. *Bull Mem Soc Anthropol Paris* **7**, 204–224 (1956).
13. Olivier, G. & Pineau, H. Olivier G., Pineau H., 1958. Détermination de l'âge du foetus et de l'embryon. *Arch. d'Anatomie* **6**, (1958).
14. Ferembach, D., Schwidetzky, I. & Stloukal, M. Recommendations pour determiner l'age et le sexe sur le squelette. *Bull Mem Soc Anthropol Paris* **6**, (1979).
15. Ferembach, D., Schwidetzky, I. & Stoukal, M. Recommendation for Age and Sex Diagnoses of Skeletons. D. Ferembach, I. Schwindezky, M. Stoukal. Journal of Human Evolution. *Journal of Human Evolution* **9**, 517–549 (1980).
16. Dittrick, J. & Suchey, J. M. Sex determination of prehistoric central California skeletal remains using discriminant analysis of the femur and humerus. *American J Phys Anthropol* **70**, 3–9 (1986).
17. France, D. L. & Horn, A. D. *Lab Manual and Workbook for Physical Anthropology*. (West Publ, St. Paul, Minn., 1992).
18. France, D. L. Observational and metric analysis of sex in the skeleton. in *Forensic osteology: advances in the identification of human remains.* 163–86 (Charles C. Thomas Publisher Ltd, Springfield, IL, 1998).
19. Iordanidis, P. Sex determination by the skeletal bones (atlas, axis, clavicle, scapula, sternum). *Ann Med Leg Criminol Police Sci Toxicol* **41**, 280–291 (1961).
20. Gualdi-Russo, E. Sex determination from the talus and calcaneus measurements. *Forensic Science International* **171**, 151–156 (2007).
21. Fontani, F. *et al.* First Bronze Age Human Mitogenomes from Calabria (Grotta Della Monaca, Southern Italy). *Genes* **12**, 636 (2021).
22. Lazaridis, I. *et al.* Genomic insights into the origin of farming in the ancient Near East. *Nature* **536**, 419–424 (2016).
23. Olalde, I. *et al.* The Beaker phenomenon and the genomic transformation of northwest Europe. *Nature* **555**, 190–196 (2018).
24. Fernandes, D. M. *et al.* The spread of steppe and Iranian-related ancestry in the islands of the western Mediterranean. *Nat Ecol Evol* **4**, 334–345 (2020).
25. Saupe, T. *et al.* Ancient genomes reveal structural shifts after the arrival of Steppe-related ancestry in the Italian Peninsula. *Current Biology* **31**, 2576-2591.e12 (2021).

26. Wilde, S. *et al.* Direct evidence for positive selection of skin, hair, and eye pigmentation in Europeans during the last 5,000 y. *Proc. Natl. Acad. Sci. U.S.A.* **111**, 4832–4837 (2014).
27. Haak, W. *et al.* Massive migration from the steppe was a source for Indo-European languages in Europe. *Nature* **522**, 207–211 (2015).
28. Narasimhan, V. M. *et al.* The formation of human populations in South and Central Asia. *Science* **365**, eaat7487 (2019).
29. Lazaridis, I. *et al.* The genetic history of the Southern Arc: A bridge between West Asia and Europe. *Science* **377**, eabm4247 (2022).
30. Yu, H. *et al.* Genomic and dietary discontinuities during the Mesolithic and Neolithic in Sicily. *iScience* **25**, 104244 (2022).
31. Rohland, N. *et al.* Three assays for in-solution enrichment of ancient human DNA at more than a million SNPs. *Genome Res.* **32**, 2068–2078 (2022).
32. Harney, É., Patterson, N., Reich, D. & Wakeley, J. Assessing the performance of qpAdm: a statistical tool for studying population admixture. *Genetics* **217**, iyaa045 (2021).
33. Patterson, N. *et al.* Large-scale migration into Britain during the Middle to Late Bronze Age. *Nature* **601**, 588–594 (2022).
34. Gretzinger, J. *et al.* Evidence for dynastic succession among early Celtic elites in Central Europe. *Nat Hum Behav* **8**, 1467–1480 (2024).
