# Supplementary material for: Archaeogenetics reconstructs demography and extreme parental consanguinity in a Bronze Age community from Southern Italy
Source: Commun Biol. 2025 Dec 15;8:1766. doi: 10.1038/s42003-025-09194-2 (PMC12706022; doi:10.1038/s42003-025-09194-2)
Supplement: Supplementary file 2 — Description of Additional Supplementary Materials [file 42003_2025_9194_MOESM2_ESM.pdf]

Description of Additional Supplementary Files

File name: Supplementary Data 1

Description: Middle Chalcolithic to Late Bronze Age palaeogenetic data from the Italian peninsula (data taken from the Allen Ancient DNA Resource repository v62)

File name: Supplementary Data 2

Description: Summary statistics from MultiQC after EAGER processing of the raw sequence data. Label "A0101" denotes Twist-enriched libraries; "A0201" is used for 1240K libraries

File name: Supplementary Data 3

Description: Contamination estimates from AuthentiCT. Label "A0201" denotes 1240K-enriched libraries

File name: Supplementary Data 4

Description: Contamination estimates after contammix

File name: Supplementary Data 5

Description: Contamination estimates after hapCon. Label "A0101" denotes Twist-enriched libraries

File name: Supplementary Data 6

Description: Results from Pairwise Mismatch Rate calculation

File name: Supplementary Data 7

Description: Haplogrep3 results

File name: Supplementary Data 8

Description: Y haplogroup identification

File name: Supplementary Data 9

Description: Kinship estimates from READv2.

File name: Supplementary Data 10

Description: Kinship estimates from KIN

File name: Supplementary Data 11

Description: ancIBD results

File name: Supplementary Data 12

Description: Runs of homozigosity segments as detected by hapROH on the individuals from Grotta della Monaca and a reference set of the most inbred prehistoric individuals found in literature

File name: Supplementary Data 13

Description: HapROH estimate of effective population size. The main Grotta_della_Monaca population is composed of individuals GMO001, GMO005, GMO006, GMO009, GMO010, GMO012, GMO015 and GMO018.

File name: Supplementary Data 14

Description: Dataset used for PCA

File name: Supplementary Data 15

Description: Results from genetic affinities tested through f4-statistics. The main GMO cluster is composed of individuals GMO001, GMO006, GMO009, GMO015, GMO018 and GMO022.

File name: Supplementary Data 16

Description: Results from individual pairwise qpWave analysis

File name: Supplementary Data 17

Description: Results from group-based f4(Mbuti, OldSteppe; X, Anatolia_N). The Grotta_della_Monaca cluster is composed of individuals GMO001, GMO005, GMO006, GMO009, GMO012, GMO015, GMO018 and GMO022.

File name: Supplementary Data 18

Description: Dataset used for qpWave and qpAdm admixture modelling

File name: Supplementary Data 19

Description: qpAdm individual distal modelling. * denotes individuals which did not fulffil SNPs cutoff, and which are only reported for comparative reasons. Base right population set is OldAfrica, WHGB, Anatolia_N and Afanasievo. Additional Levantine ancestry was tested by adding CHG/Iran_N as either left or right populations. For this test, we report the fitting model with the lower standard errors in each component.

File name: Supplementary Data 20

Description: qpAdm group-based distal modelling. * denotes individuals which did not fulffil SNPs cutoff, and which are only reported for comparative reasons. Base right population set is OldAfrica, WHGB, Anatolia_N and Afanasievo. Additional Levantine ancestry was tested by adding CHG/Iran_N as either left or right populations. For this test, we report the fitting model with the lower standard errors in each component. Additional North African ancestry was tested by adding OldNorthAfrican as source population and Morocco_Iberomaurusian.AG as a right population, but not found in any of the tested population.

File name: Supplementary Data 21

Description: qpAdm proximal modelling

File name: Supplementary Data 22

Description: Screening results of SNPs associated with phenotypic traits and monogenic diseases. For each individual, SNPs coverage are reported as number of ancestral reads / number of derived reads

File name: Supplementary Data 23

Description: HirisPlex Phenotype prediction
