# Supplementary material for: Archaeogenetics reconstructs demography and extreme parental consanguinity in a Bronze Age community from Southern Italy
Source: Commun Biol. 2025 Dec 15;8:1766. doi: 10.1038/s42003-025-09194-2 (PMC12706022; doi:10.1038/s42003-025-09194-2)
Supplement: Supplementary file 4 — Reporting Summary [file 42003_2025_9194_MOESM4_ESM.pdf]

nature portfolio

Corresponding author(s): Francesco Fontani

Last updated by author(s): Oct 24, 2025

Reporting Summary

Nature Portfolio wishes to improve the reproducibility of the work that we publish. This form provides structure for consistency and transparency in reporting. For further information on Nature Portfolio policies, see our Editorial Policies and the Editorial Policy Checklist.

Statistics

For all statistical analyses, confirm that the following items are present in the figure legend, table legend, main text, or Methods section.

n/a	Confirmed	
☐	☒	The exact sample size (*n*) for each experimental group/condition, given as a discrete number and unit of measurement
☐	☒	A statement on whether measurements were taken from distinct samples or whether the same sample was measured repeatedly
☒	☐	The statistical test(s) used AND whether they are one- or two-sided<br>*Only common tests should be described solely by name; describe more complex techniques in the Methods section.*
☒	☐	A description of all covariates tested
☒	☐	A description of any assumptions or corrections, such as tests of normality and adjustment for multiple comparisons
☐	☒	A full description of the statistical parameters including central tendency (e.g. means) or other basic estimates (e.g. regression coefficient) AND variation (e.g. standard deviation) or associated estimates of uncertainty (e.g. confidence intervals)
☐	☒	For null hypothesis testing, the test statistic (e.g. *F*, *t*, *r*) with confidence intervals, effect sizes, degrees of freedom and *P* value noted<br>*Give P values as exact values whenever suitable.*
☒	☐	For Bayesian analysis, information on the choice of priors and Markov chain Monte Carlo settings
☒	☐	For hierarchical and complex designs, identification of the appropriate level for tests and full reporting of outcomes
☒	☐	Estimates of effect sizes (e.g. Cohen's *d*, Pearson's *r*), indicating how they were calculated

Our web collection on statistics for biologists contains articles on many of the points above.

Software and code

Policy information about availability of computer code

Data collection: na

Data analysis: na

For manuscripts utilizing custom algorithms or software that are central to the research but not yet described in published literature, software must be made available to editors and reviewers. We strongly encourage code deposition in a community repository (e.g. GitHub). See the Nature Portfolio guidelines for submitting code & software for further information.

Data

Policy information about availability of data

All manuscripts must include a data availability statement. This statement should provide the following information, where applicable:

- Accession codes, unique identifiers, or web links for publicly available datasets
- A description of any restrictions on data availability
- For clinical datasets or third party data, please ensure that the statement adheres to our policy

The raw sequencing data (FASTQ files) generated in this study are publicly available in the European Nucleotide Archive (ENA: https://www.ebi.ac.uk/ena/browser/home) under project accession number PRJEB94318. The osteological remains are under the protection of the Soprintendenza Archeologia, Belle Arti e Paesaggio of the Cosenza province. They are formally deposited at the municipal deposits in Sant'Agata d'Esaro (protocol number 11710-P, 5/12/2023).

Research involving human participants, their data, or biological material

Policy information about studies with human participants or human data. See also policy information about sex, gender (identity/presentation), and sexual orientation and race, ethnicity and racism.

Reporting on sex and gender	Given that the analysed specimens originate from ancient humans material, we used the term sex throughout the text to indicate biological sex.
Reporting on race, ethnicity, or other socially relevant groupings	Group-related inferences were made through archaeological and statistical inferences of the genetic material.
Population characteristics	This does not apply to our archaeoanthropological material.
Recruitment	This does not apply to our archaeoanthropological material.
Ethics oversight	The Soprintendenza Archeologia Belle Arti e Paesaggio per le province di Catanzaro, Crotone e Cosenza provided permits for the study of the anthropological material presented in this study (protocol number 0015731, 24/12/2018)

Note that full information on the approval of the study protocol must also be provided in the manuscript.

Field-specific reporting

Please select the one below that is the best fit for your research. If you are not sure, read the appropriate sections before making your selection.

☒ Life sciences ☐ Behavioural & social sciences ☐ Ecological, evolutionary & environmental sciences

For a reference copy of the document with all sections, see nature.com/documents/nr-reporting-summary-flat.pdf

Life sciences study design

All studies must disclose on these points even when the disclosure is negative.

Sample size	All available petrous bones and additional teeth were selected, the only after visually assessing their preservation, specifically including only intact specimens that showed no signs of breakage or significant fracturing. We relied on anthropological analysis to establish a minimum number of individuals (n=21). The number of selected bones broadly overlaps with the expected number of individuals buried in the cavity.
Data exclusions	We only excluded from our analyses samples which reported too low preservation of the endogenous material
Replication	The produced genetic data are made available on public and open repository to ensure replicability
Randomization	We assessed cladality and genetic affinities of samples through statistical inferences, as extensively described in the methods section
Blinding	Despite the commingled nature of the remains, and the large corpus of archaeological and radiocarbon information from the cavity, the authors relied on statistical and population genetics analyses to prevent interpretative bias during the research

Reporting for specific materials, systems and methods

We require information from authors about some types of materials, experimental systems and methods used in many studies. Here, indicate whether each material, system or method listed is relevant to your study. If you are not sure if a list item applies to your research, read the appropriate section before selecting a response.

Materials & experimental systems

n/a	Involved in the study	
☒	☐	Antibodies
☒	☐	Eukaryotic cell lines
☐	☒	Palaeontology and archaeology
☒	☐	Animals and other organisms
☒	☐	Clinical data
☒	☐	Dual use research of concern
☒	☐	Plants

Methods

n/a	Involved in the study	
☒	☐	ChIP-seq
☒	☐	Flow cytometry
☒	☐	MRI-based neuroimaging

Palaeontology and Archaeology

Specimen provenance	All the samples come from the archaeological site of Grotta della Monaca. The osteological remains are under the protection of the Soprintendenza Archeologia Belle Arti e Paesaggio per le province di Catanzaro, Crotone e Cosenza, which provided permits for the study to the University of Bologna (professor Donata Luiselli, protocol number 0015731, 24/12/2018)
Specimen deposition	The osteological remains are formally deposited at the municipal deposits in Sant'Agata d'Esaro (protocol number 11710-P, 5/12/2023).
Dating methods	For the two individuals specifically dated for this study after palaeogenetic analysis, collagen extraction from the samples was performed using the Longin method at the chemical laboratories of the CEDAD (Lecce, Italy).The selected collagen fraction was converted into CO_2 by combustion in sealed quartz tubes containing CuO and silver wool. The CO_2 was then reduced to graphite at 600°C using hydrogen gas and iron powder as a catalyst. The AMS radiocarbon analyses were conducted using a 3 MV Tandetron accelerator (High Voltage Engineering Europa BV, model 4130HC). The obtained $^{14}C/^{12}C$ ratios were corrected for isotopic fractionation based on $\delta^{13}C$ values measured directly by the AMS system, as well as for background contributions from both the instrument and the chemical preparation. Conventional radiocarbon ages were computed following the method by Stuiver and Polach, and calibrated using the latest IntCal20 calibration curve.

☒ Tick this box to confirm that the raw and calibrated dates are available in the paper or in Supplementary Information.

Ethics oversight	The osteological remains are under the protection of the Soprintendenza Archeologia Belle Arti e Paesaggio per le province di Catanzaro, Crotone e Cosenza, which provided permits for the study to the University of Bologna (professor Donata Luiselli, protocol number 0015731, 24/12/2018). No ethical guidance is necessary to generate genomic information from archaeoanthropological material in Italy

Note that full information on the approval of the study protocol must also be provided in the manuscript.

Plants

Seed stocks	na
Novel plant genotypes	na
Authentication	na
